# Supplementary material for: Risk of 30-day hospital readmission associated with medical conditions and drug regimens of polymedicated, older inpatients discharged home: a registry-based cohort study
Source: BMJ Open. 2021 Jul 14;11(7):e052755. doi: 10.1136/bmjopen-2021-052755 (PMC8281082; doi:10.1136/bmjopen-2021-052755)
Supplement: Supplementary data [file bmjopen-2021-052755supp001.pdf]

Supplementary table. Descriptive statistics of drugs prescribed per hospital stays (N = 13,802) at discharge based on the ATC Classification System.

**Drug classes based on the ATC Classification System**	**Min-Max**	**Mean (SD)**
First level, anatomical main group		
Blood and blood forming organs (B)	0–5	1.15 (0.86)
Dermatologicals (D)	0–3	0.04 (0.21)
Genitourinary system and sex hormones (G)	0–4	0.21 (0.47)
Systemic hormonal preparations, excl. sex hormones and insulins (H)	0–4	0.20 (0.46)
Anti-infectives for systemic use (J)	0–4	0.24 (0.47)
Antineoplastic and immunomodulating agents (L)	0–5	0.05 (0.23)
Musculo-skeletal system (M)	0–3	0.15 (0.39)
Antiparasitic products, insecticides and repellents (P)	0–2	0.02 (0.13)
Respiratory system (R)	0–7	0.28 (0.72)
Sensory organs (S)	0–6	0.10 (0.39)
Second level, therapeutic subgroup		
Stomatological preparations (A01)	0–1	0.00 (0.06)
Drugs for acid-related disorders (A02)	0–3	0.56 (0.52)
Drugs for functional gastrointestinal disorders (A03)	0–3	0.07 (0.28)
Antiemetics and antinauseants (A04)	0–1	0.01 (0.08)
Bile and liver therapy (A05)	0–1	0.00 (0.05)
Drugs for constipation (A06)	0–3	0.15 (0.40)
Antidiarrhoeals, intestinal anti-inflammatory/anti-infective agents (A07)	0–2	0.03 (0.18)
Digestives, incl. Enzymes (A09)	0–2	0.02 (0.13)
Drugs used in diabetes (A10)	0–5	0.26 (0.63)
Vitamins (A11)	0–4	0.15 (0.44)
Mineral supplements (A12)	0–3	0.29 (0.51)
Other alimentary tract and metabolism products (A16)	0–1	0.00 (0.05)
Cardiac therapy drugs (C01)	0–4	0.14 (0.42)
Antihypertensives (C02)	0–2	0.02 (0.17)
Diuretics (C03)	0–3	0.27 (0.53)
Peripheral vasodilators (C04)	0–1	0.00 (0.06)
Vasoprotectives (C05)	0–3	0.02 (0.14)
Beta-blocking agents (C07)	0–2	0.46 (0.51)
Calcium channel blockers (C08)	0–2	0.16 (0.37)
Agents acting on the renin-angiotensin system (C09)	0–3	0.64 (0.62)
Lipid modifying agents (C10)	0–3	0.43 (0.52)
Anaesthetics (N01)	0–1	0.00 (0.05)
Analgaesics (N02)	0–7	1.02 (0.91)
Antiepileptics (N03)	0–5	0.11 (0.35)
Drugs for Parkinson's disease (N04)	0–5	0.04 (0.24)
Psycholeptics (N05)	0–6	0.53 (0.73)
Psychoanaleptics (N06)	0–3	0.20 (0.44)
Other nervous system drugs(N07)	0–3	0.03 (0.19)
**Total number of drugs**	**5–30**	**8.95 (3.24)**
