# Supplementary material for: Risk of 30-day hospital readmission associated with medical conditions and drug regimens of polymedicated, older inpatients discharged home: a registry-based cohort study
Source: BMJ Open. 2021 Jul 14;11(7):e052755. doi: 10.1136/bmjopen-2021-052755 (PMC8281082; doi:10.1136/bmjopen-2021-052755)
Supplement: Author's manuscript [file bmjopen-2021-052755.draft_revisions.pdf]

BMJ Open is committed to open peer review. As part of this commitment we make the peer review history of every article we publish publicly available.

When an article is published we post the peer reviewers' comments and the authors' responses online. We also post the versions of the paper that were used during peer review. These are the versions that the peer review comments apply to.

The versions of the paper that follow are the versions that were submitted during the peer review process. They are not the versions of record or the final published versions. They should not be cited or distributed as the published version of this manuscript.

BMJ Open is an open access journal and the full, final, typeset and author-corrected version of record of the manuscript is available on our site with no access controls, subscription charges or pay-per-view fees (http://bmjopen.bmj.com).

If you have any questions on BMJ Open's open peer review process please email info.bmjopen@bmj.com

BMJ Open

A registry-based cohort study of disease- and drug-related 30-day hospital readmissions among polymedicated older inpatients

Journal:	*BMJ Open*
Manuscript ID	bmjopen-2021-052755
Article Type:	Original research
Date Submitted by the Author:	25-Apr-2021
Complete List of Authors:	Pereira, Filipa; University of Porto, Institute of Biomedical Sciences Abel Salazar; HES-SO University of Applied Sciences and Arts Western Switzerland, School of Health Sciences<br>Verloo, Henk; HES-SO University of Applied Sciences and Arts Western Switzerland, School of Health Sciences; Lausanne University Hospital, Service of Old Age Psychiatry<br>Zhivko, Taushanov; University of Geneva<br>Di Giovanni, Saviana; HES-SO University of Applied Sciences and Arts Western Switzerland, School of Health Sciences; Pharmacy Benu Tavil-Chatton<br>Meyer-Massetti, Carla; University of Bern, Institute for Primary Health Care<br>Von-Gunten, Armin; Lausanne University Hospital, Service of Old Age Psychiatry<br>Martins, Maria Manuela; Porto Higher School of Nursing; University of Porto, Institute of Biomedical Sciences Abel Salazar<br>Wernli, Boris; University of Lausanne, FORS, Swiss Centre of Expertise in the Social Sciences
Keywords:	Health informatics < BIOTECHNOLOGY & BIOINFORMATICS, GERIATRIC MEDICINE, CLINICAL PHARMACOLOGY, EPIDEMIOLOGY

SCHOLARONE™
Manuscripts

I, the Submitting Author has the right to grant and does grant on behalf of all authors of the Work (as defined in the below author licence), an exclusive licence and/or a non-exclusive licence for contributions from authors who are: i) UK Crown employees; ii) where BMJ has agreed a CC-BY licence shall apply, and/or iii) in accordance with the terms applicable for US Federal Government officers or employees acting as part of their official duties; on a worldwide, perpetual, irrevocable, royalty-free basis to BMJ Publishing Group Ltd ("BMJ") its licensees and where the relevant Journal is co-owned by BMJ to the co-owners of the Journal, to publish the Work in this journal and any other BMJ products and to exploit all rights, as set out in our licence.

The Submitting Author accepts and understands that any supply made under these terms is made by BMJ to the Submitting Author unless you are acting as an employee on behalf of your employer or a postgraduate student of an affiliated institution which is paying any applicable article publishing charge ("APC") for Open Access articles. Where the Submitting Author wishes to make the Work available on an Open Access basis (and intends to pay the relevant APC), the terms of reuse of such Open Access shall be governed by a Creative Commons licence – details of these licences and which Creative Commons licence will apply to this Work are set out in our licence referred to above.

Other than as permitted in any relevant BMJ Author's Self Archiving Policies, I confirm this Work has not been accepted for publication elsewhere, is not being considered for publication elsewhere and does not duplicate material already published. I confirm all authors consent to publication of this Work and authorise the granting of this licence.

For peer review only

A registry-based cohort study of disease- and drug-related 30-day hospital readmissions among polymedicated older inpatients

---

Pereira Filipa[1], Verloo Henk[2], Taushanov Zhivko[3], Di Giovanni Saviana[4], Meyer-Massetti Carla[5], von Gunten Armin[6], Martins Maria Manuela[7], Wernli Boris[8]

[1] Institute of Biomedical Sciences Abel Salazar, University of Porto
Rua de Jorge Viterbo Ferreira, 228 4050-313 Porto, Portugal
School of Health Sciences, HES-SO University of Applied Sciences and Arts Western Switzerland
5, Chemin de l'Agasse, CH-1950 Sion, Switzerland
Email: filipa.pereira@hevs.ch; Phone: +41 78 666 17 00; Fax: +41 27 606 84 00
ORCID: https://orcid.org/0000-0001-9207-4856

[2] School of Health Sciences, HES-SO University of Applied Sciences and Arts Western Switzerland
5, Chemin de l'Agasse, CH-1950 Sion, Switzerland
Service of Old Age Psychiatry, Lausanne University Hospital
Email: henk.verloo@hevs.ch; Phone: +41 27 606 84 34
ORCID: http://orcid.org/0000-0002-5375-3255

[3] University of Geneva
CH-1205 Geneva, Switzerland
Email: zhivko.taushanov@unige.ch
ORCID: https://orcid.org/0000-0002-3798-757X

[4] School of Health Sciences, HES-SO University of Applied Sciences and Arts Western Switzerland
5, Chemin de l'Agasse, CH-1950 Sion, Switzerland
Pharmacy Benu Tavil-Chatton
Grand rue 11, CH-1110 Morges, Switzerland
Email: saviana.digiovanni@gmail.com

[5] Institute for Primary Health Care, University of Bern
Mittelstrasse 43, CH-3012 Bern, Switzerland
Email: carla.meyer-massetti@biham.unibe.ch
ORCID: https://orcid.org/0000-0002-3523-5729

[6] Service of Old Age Psychiatry,
Lausanne University Hospital
Route de Cery 60, 1008 Prilly, Switzerland
Email: armin.von-gunten@chuv.ch; Phone: +41 21 314 52 67

For peer review only

ORCID: https://orcid.org/0000-0001-7852-3803

[7] Higher School of Nursing of Porto
Institute of Biomedical Sciences Abel Salazar, University of Porto
Rua Dr. António Bernardino de Almeida
4200-072 Porto, Portugal
Email: mmartins@esenf.pt; Phone: +351 22 507 35 00
ORCID: https://orcid.org/0000-0003-1527-9940

[8] FORS, Swiss Centre of Expertise in the Social Sciences, University of Lausanne
Géopolis, CH-1015 Lausanne, Switzerland
Email: boris.wernli@fors.unil.ch; Phone: +41 21 692 37 23
ORCID: https://orcid.org/0000-0002-5567-1317

Corresponding author:
Pereira F
Institute of Biomedical Sciences Abel Salazar, University of Porto
Rua de Jorge Viterbo Ferreira, 228 4050-313 Porto, Portugal
School of Health Sciences, HES-SO University of Applied Sciences and Arts Western Switzerland
5, Chemin de l'Agasse, CH-1950 Sion, Switzerland
Email: filipa.pereira@hevs.ch; Phone: +41 78 666 17 00; Fax: +41 27 606 84 00
ORCID: https://orcid.org/0000-0001-9207-4856

For peer review only

Abstract

Objectives: The present study analysed four years of a hospital register (2015–2018) to investigate associations between 30-day hospital readmission risk and the medical conditions and drug regimens of polymedicated, older inpatients discharged home.

Design: Population-based longitudinal study.

Setting: A public general hospital centre in the French-speaking part of Switzerland.

Participants: We explored the electronic records of 20,422 inpatient stays by polymedicated, home-dwelling older adults held in the hospital's patient register. We identified 13,802 separate patients over 64 years old.

Outcome measures: Sociodemographic characteristics, medical conditions, and drug regimen data associated with readmission within 30 days of discharge.

Results: The overall 30-day hospital readmission rate was 7.8%. Higher risks of hospital readmission were associated with longer hospital length of stay (OR = 1.014), impaired mobility (OR = 1.218), multimorbidity (OR = 1.419), tumoural disease (OR = 2.538), polypharmacy (OR = 1.043), and certain specific drugs, including antiemetics and antinauseants (OR = 3.216), antihypertensives (OR = 1.771), drugs for functional gastrointestinal disorders (OR = 1.424), systemic hormonal preparations (OR = 1.207), and vitamins (OR = 1.201), as well as the concurrent use of beta-blocking agents and proton pump inhibitors (OR = 1.367).

Conclusions: Thirty-day hospital readmission risks were associated with longer hospital length of stay, health disorders, polypharmacy and drug regimens. The drug regimen patterns increasing the risk of hospital readmission were very heterogeneous. Further research is needed to explore hospital readmissions caused solely by specific drugs and drug–drug interactions.

Keywords: polypharmacy; odds ratio; logistic regression; hospital register; ATC Classification System; adverse-drug events; hospital readmission.

Strengths and limitations of this study:

- The records of 20,422 separate hospitalisations involving 13,802 polymedicated home-dwelling older patients were investigated for the prevalence of 30-day hospital readmission.
- The study includes four years' data from an exhaustive hospital register (2015–2018).
- A whole series of sociodemographic and clinical parameters, medical conditions and prescribed drugs were used to predict the probability of hospital readmission.
- Analyses were correlational and no causality could be demonstrated.
- Although the study considered statistical associations between drugs and rehospitalisations, it did not use clinically diagnosed drug–drug interactions.

For peer review only - http://bmjopen.bmj.com/site/about/guidelines.xhtml

Introduction

Longitudinal studies have demonstrated that approximately 20% of the home-dwelling older adults supported by home health-care services experienced hospital readmission within 30 days of their discharge (1-3). For many older adults, readmission to an acute hospital is associated with a functional decline that has not always recovered by the time they are discharged (4). However, the systematic review by Hansen *et al*. revealed wide-ranging estimates (5%–79%) of how many hospital readmissions were preventable (5). The period between hospital discharge and readmission has not always been clearly stated in the literature, ranging from 30 days to 3 years. However, 30 days is the most frequently used in public health policy when measuring health-care system performance (6-8).

Numerous determinants have been identified and associated with hospital readmissions, e.g. sociodemographic and individual characteristics, multimorbidity and medical events (9, 10). A substantial risk of 30-day hospital readmission has been associated with older inpatients treated for different diseases and surgical interventions involving hip fracture, cancer, bypass, acute cardiovascular events or complex surgery (11). The reasons for hospital readmission after a surgical intervention are often not directly related to the surgery itself but rather to underlying chronic health conditions (12). Thus, chronic diseases may play an important role in readmission risk, independently of the reason for the initial hospitalisation (13, 14). Chronic diseases are not isolated health conditions among older inpatients: they can influence each other and treatment for one disease may adversely affect another (15). For all these reasons, patterns of 30-day hospital readmissions may be very complex (16).

Multimorbidity, in the case of two or more diseases (17, 18), may require taking multiple medicines (19), known as polypharmacy (PP) when daily intake involves five or more drugs (20). Increasing incidences of multimorbidity with age, and consequently PP, add to the complexity of managing older inpatients' drug prescriptions, particularly at hospital discharge (21, 22). PP and inadequate drug management are significant risk factors for adverse drug events (ADEs)—the most common post-discharge complications—alongside hospital-acquired infections and procedural complications (23, 24). ADEs resulting from inappropriate drug prescribing, discrepancies between prescribed and current regimens, poor adherence and the inadequate surveillance of adverse effects frequently lead to hospital admissions, readmissions (8) and other undesirable consequences such as increased morbidity, decreased autonomy, institutionalisation and even early death (25, 26). A systematic review by Morabet *et al*. indicated ADE rates of 18%–38% after hospital discharge and 4.5%–24% hospital readmission rates due to those ADEs (27). Because older adults use more drugs, they are at a greater risk of drug-related readmission. Numerous studies have found that nearly 30% of older inpatients experienced ADEs within three weeks of hospital discharge, almost three-quarters of which could have been prevented or lessened (10, 28, 29).

Despite the significant overall impact of ADEs on hospital readmission rates, little is known about hospital readmission risk's associations with medical conditions and drug regimens (30, 31). Morabet *et al*. revealed the high prevalence of antibiotics, diuretics, vitamin K antagonists, opioids, antidiabetics,

For peer review only

For peer review only - http://bmjopen.bmj.com/site/about/guidelines.xhtml

anti-cancer drugs, antihypertensives, digitalis glycosides, corticosteroids and psychotropic drugs in drug-related hospital readmissions (27). Samoy *et al*. reported that anticoagulants, hypoglycaemics, beta-blocking agents, antidepressants, calcium channel blockers and lenograstim were associated with high risks of hospital readmission (32). A retrospective patient record study by Teymoorian *et al*. reported that anticoagulants and antiplatelet agents, diuretics and antihypertensives, and opioids were associated with a high risk of persons aged 80 years old or more being readmitted to hospital within 30 days (33). Blanc *et al*. reported the readmission scores of different drugs in a large sample of 10,374 adult hospital admissions in general medicine. Taking beta-blocking agents, calcium channel blockers, diuretics, hypoglycaemic drugs or opioids was a significant risk for 30-day readmission (9).

Besides higher risks of drug-related hospital readmission, some studies have also investigated associations between combining drugs—a common practice when treating complex diseases or co-existing medical conditions—and drug-related hospital readmissions. Although using multiple drugs may be good clinical practice and compliant with guidelines for treating certain diseases, one significant consequence of combining drugs is that patients face much higher risks of ADEs, which can be caused by drug–drug interactions (34-36). ADEs can emerge because a drug's pharmacokinetics and pharmacodynamics change if taken with another drug (36). Moura *et al*. found that participants with potential drug–drug interactions on their prescription list had a 2.4 times higher adjusted odds ratio (OR) of being readmitted (37).

Even though some studies have reported high numbers of readmissions among home-dwelling older patients for a variety of drugs (38), this health issue was mostly investigated using prospective or cross-sectional studies with small samples. More insight is needed into patterns of drug-related hospital readmissions and risk factors in order to design better interventions for addressing ADEs (39, 40). As part of a broader project (41), the present study's goal was to use hospital register data to prioritise risk factors for hospital readmission. We hypothesised that sociodemographic characteristics, medical conditions (defined using the WHO's International Classification of Diseases, tenth revision: ICD-10, and the Swiss Classification of Surgical Interventions: CHOP), and drug prescriptions (based on the WHO's Anatomical Therapeutic Chemical (ATC) Classification System) were significant risk factors for 30-day hospital readmission for discharged older adults.

Material and Methods

Study Design

This population-based longitudinal study investigated the associations between the risks of discharged, polymedicated, home-dwelling older patients being readmitted to hospital within 30 days and their drug prescriptions and medical conditions. The study was performed with close regard to the REporting of studies Conducted using Observational Routinely collected health Data (RECORD) statement (42).

For peer review only - http://bmjopen.bmj.com/site/about/guidelines.xhtml

Population and Data Collection

Our custom, four-year, population-based dataset was composed of polymedicated (five or more drugs prescribed at hospital discharge), multimorbid (two or more ICD-10 diagnoses), home-dwelling older adult participants (65 years old and above) admitted and readmitted to a public general hospital in the French-speaking part of Switzerland. This specific population was selected because of its increased risk of hospital readmission (10, 28, 29). The hospital register contains a comprehensive and exhaustive electronic health record (43). However, no electronic patient records were available for adult psychiatry for 2015–2018. The extracted patient data contained sociodemographic characteristics, medical and surgical diagnoses, routinely assessed clinical data (such as gait, falls risk or hearing) from hospitalised patients with at least five prescribed drugs and their prescribed drugs at discharge. Medical and surgical diagnoses were coded based on the ICD-10 and CHOP (44). Drug classification was based on the WHO's ATC Classification System (45).

The strategy for transforming and synthesising the data extracted from the register's multiple dataset sources was based on Olsen's register-based methodological considerations (46) and has been documented elsewhere (47). Our dataset was composed of 20,422 hospital admission records running from January 2015 to December 2018, with similar numbers of annual hospital admissions: 5134, 5095, 5125, and 5068, respectively.

Patient and Public Involvement

Neither patients nor public were directly involved in the development of the research questions, study design, outcome measures, recruitment and conduct of the study.

Dataset Customisation for Predictive Analysis

The dataset was recoded and customised to identify the frequency of older patients' hospital admissions. Each subject's unique identifier was used to distinguish their different hospital stays from 2015–2018. The dataset included 13,802 hospital stays involving older inpatients discharged home and whose data were complete (no missing values). These complete datasets related to 8,878 different individuals, with an average of 1.55 inpatient hospital stays. Sociodemographic and clinical data were considered independent variables and used to compute the predictive models. Readmission following discharge home was defined as the dependent variable of interest and was dichotomised (0 = no, 1 = yes) based on 30-day readmission between 2015 and 2018. Furthermore, the custom dataset was composed of six clinical clusters based on agglomerative hierarchical clustering methods for identifying clinically relevant characteristics and representing older inpatients' health status. Medical status and drugs data were recoded and copied to an exploitable population database (47).

Sociodemographic Variables and Length of Stay

Sex and age were included in the analysis as sociodemographic control variables. The total sample's mean age was 77.77 years old (SD = 7.48), and 57% were women. Age was considered a continuous

For peer review only - http://bmjopen.bmj.com/site/about/guidelines.xhtml

variable as its progressive impact has been proven in preliminary investigations and previous studies (48). The average hospital length of stay (LOS) was 8.44 days (SD = 7.58).

Health Variables

Numerous variables were used to describe older patients' health status during each hospital stay. Three of six preliminarily computed hierarchical clusters (47) were included in the modeling analysis as confounding variables: the mobility cluster, the dependency in the activities of daily living cluster and the mental state cluster (47). Twenty-five per cent of the sample had impaired mobility, 4% were impaired in their activities of daily living and 4% showed mental impairment at discharge. Our sample population averaged 4.58 (SD = 0.92) ICD-10 diagnoses, with their individual numbers used to model multimorbidity, and 1.80 (SD = 1.76) surgical interventions (CHOP) performed during the hospitalisation. The selected medical diagnoses distinguished patients affected by circulatory (24%), infectious (3%), and respiratory diseases (11%), as well as trauma (8%) and tumours (11%). Finally, the year of hospitalisation was introduced as a control variable, based on the fact that earlier admission to hospital during this period led to a higher probability of unplanned readmissions during the entire period covered.

Included Drugs

Drugs were classified according to the WHO's ATC Classification System (49) and then included in the predictive model as independent variables. To ensure robust statistical results, the model only included drug categories prescribed to at least 30 participants (n = 13,802). Drug prescription was considered continuous, with an average of 8.95 (SD = 3.24) drugs per patient prescribed at hospital discharge. Table 1 presents the prescribed ATC classified drugs included in the predictive model as independent variables.

[Insert Table 1]

Data analysis strategy

Data were extracted into a Microsoft Excel spreadsheet (Microsoft, Redmond, Washington, United States) and then imported into SPSS software, version 26.0 (IBM Corp, Armonk, New York, United States). We examined statistical associations between hospital readmissions and patient age and sex, LOS, principal and related ICD-10 diagnoses, CHOP interventions and drug prescriptions during hospitalisations. A causality analysis between those variables was impossible given our retrospective data collection method, our inability to calculate the time between drug intake and readmission, and the potential drug changes between hospitalisation sequences. We conducted a bivariate analysis relating the independent variables to 30-day readmission after discharge home from 2015–2018. In a second stage, a series of multilevel binary logistic regression models were computed to estimate how sets of predictors influenced the probability of 30-day hospital readmission. The first level concerned hospital LOS, and the second level included individuals' characteristics and health conditions. The model projected each

For peer review only - http://bmjopen.bmj.com/site/about/guidelines.xhtml

predictor's impact, other things being equal, by estimating its net impact, controlling for other factors (adjusted ORs). The model also considered correlations between each subject's different variables, which were generally not independent (50). The model's random intercept design allowed each individual's intercept to vary, assuming that some unmeasured traits remained stable over time and allowing a better estimation of the model's parameters. The other estimated parameters, on the other hand, had the same effect on every subject. This regression analysis dichotomised the probabilities of future readmissions within 30 days (0 = no, 1 = yes) of discharge home. Since the data were based on a whole population, not a sample, the ORs' confidence intervals and statistical tests were used to indicate the robustness of relationships (they usually only make sense for statistical inference).

Patients and public involvement

Patients were not involved in the development of the research questions, study design, outcome measures and conduct of the study.

Results

Associations between Thirty-day Hospital Readmission and Sociodemographic Characteristics and Medical Conditions

The prevalence of 30-day hospital readmission for older patients discharged home was 7.8%. Bivariate associations showed significant differences between participants' sociodemographic characteristics and medical conditions (Table 2). Men showed a slightly higher proportion of 30-day hospital readmissions than women (8.2% vs 7.3%). However, age did not significantly affect the probability of 30-day readmission. More readmissions were also seen among older patients with a circulatory disease (8.2% vs 6.5%), those not affected by trauma (8.0% vs 5.8%), and especially those with a tumour (15.1% vs 6.9%). Multimorbidity also increased the prevalence of 30-day hospital readmissions—from 1.5% for older patients with a single ICD-10 condition to 8.8% for those with five.

[Insert Table 2]

Associations between Thirty-day Hospital Readmission and Drugs

On average, older patients readmitted within 30 days had more prescribed drugs than those who were not readmitted (9.95 drugs vs 8.87). We found a linear relationship between the 30-day readmission rate and the average number of prescribed drugs ($p > 0.001$), which supported the absence of a cut-off point in this relationship (Figure 1).

[Insert Figure 1]

Among the most robust statistical associations with 30-day hospital readmissions involved the classes of drugs including antineoplasics and immunomodulators (12.6% vs 7.6% for those not treated with them) and taking antiemetics and antinauseants (27.7% vs 7.7%). There was also a high prevalence of 30-day hospital readmission among participants taking drugs for functional gastrointestinal disorders (13.4% vs 7.4%) and antihypertensives (14.1% vs 7.7%) (Table 3).

For peer review only - http://bmjopen.bmj.com/site/about/guidelines.xhtml

For peer review only

[Insert Table 3]

Baseline Multivariate Model

A baseline, multivariate logistic regression model including older patients' sociodemographic and clinical variables, but not their prescribed drugs at discharge, was computed to predict 30-day hospital readmission after discharge home (Table 4). Neither sex nor age had a significant impact. On the contrary, LOS had a significant impact (OR = 1.014 for each additional day; 95% CI: 1.006–1.021), as did mobility (OR = 1.218 for older patients with an impaired mobility status; 95% CI: 1.039–1.427). Dependence in the activities of daily living and mental health status showed no influence. Concerning diagnoses measured in the ICD-10, we found that older patients with a tumoural disease (OR = 2.538; 95% CI: 2.089–3.082) were much more susceptible to 30-day hospital readmission. Patients with circulatory pathologies showed no difference from the reference category (OR = 0.938; 95% CI: 0.783–1.124), and nor did those with respiratory problems (OR = 1.100; 95% CI: 0.875–1.382), trauma (OR = 0.847; 95% CI: 0.633–1.134) or infection-related problems (OR = 1.381; 95% CI: 0.964–1.977; $p = 0.078$). Having several pathologies predicted a higher probability of readmission (OR = 1.419 per additional ICD-10 condition; 95% CI: 1.282–1.572), whereas the number of surgical procedures had no noticeable impact (OR = 0.978; 95% CI: 0.938–1.020). The year of hospital stay did have an impact, however, as the earlier the hospitalisation during the four years under review, the higher the probability of readmission (OR = 0.933 per additional year; 95% CI: 0.880–0.990).

[Insert Table 4]

Prediction of 30-day Hospital Readmission and Drug Prescriptions

Table 5 presents the baseline logistic regression model completed with the drugs prescribed to older patients at discharge home that were significantly associated ($p = < 0.05$) with 30-day hospital readmission. It was impossible to introduce the total number of drugs prescribed jointly in this model because of their collinearity with other drug variables. Non-significant drugs and other variables have been omitted from Table 3 in order to simplify the presentation. The probabilities of 30-day hospital readmission are presented in descending order of discharged older patients' ORs for each additional unit of the drugs in question. Intake of antiemetics and antinauseants was very strongly linked to 30-day readmission (OR = 3.216 for each additional drug unit taken; 95% CI: 1.842–5.617), as were those of antihypertensives (OR = 1.771; 95%CI: 1.287–2.438), gastrointestinal drugs (OR = 1.424; 95% CI: 1.166–1.739), systemic hormonal preparations (OR = 1.207; 95% CI: 1.052–1.385) and vitamins (OR = 1.201; 95% CI: 1.049–1.374). On the contrary, the intake of lipid-modifying agents was associated with a decrease in 30-day hospital readmissions (OR = 0.841 for each drug from this class prescribed; 95% CI: 0.732–0.967).

[Insert Table 5]

Drug Interactions and 30-day Hospital Readmissions

For peer review only - http://bmjopen.bmj.com/site/about/guidelines.xhtml

For statistical purposes, drug–drug interactions between different ATC drug classes (49) were operationalised as dichotomised variables (0 = no simultaneous use of drugs from both classes, 1 = simultaneous use of drugs from both classes) and added to the previous model. Drug class interactions were selected based on a literature review, significant ORs, and expert opinions (51). The model considered drug class interactions for the: 1) cardiovascular system * central nervous system, gastrointestinal system, and metabolism * cardiovascular system; 2) gastrointestinal system and metabolism * central nervous system; 3) cardiovascular system * anti-infectives; and 4) central nervous system * anti-infectives. The analysis was carried out controlling for the basic model's variables (Table 4), and the table reports the ORs for each additional unit of the statistically significant drugs in question, as well as for significant drug interactions. Antiemetics and antinauseants were very strongly associated with 30-day readmission (OR = 3.222; 95% CI: 1.844–5.630), as were drugs regulating the gastrointestinal tract (OR = 1.428; 95% CI: 1.169–1.744) and systemic hormones (OR = 1.210; 95% CI: 1.054–1.390). The joint intake of beta-blocking agents and drugs for acid-related disorders was significantly associated with 30-day hospital readmission (OR = 1.367; 95% CI: 1.046–1.788); this is the only significant drug interaction in Table 4. On the contrary, lipid-modifying agents were associated with lower 30-day hospital readmission (OR = 0.838), as were substances acting on the renin–angiotensin system (OR = 0.892; 95% CI: 0.796–0.999) (Table 6).

[Insert Table 6]

Discussion

The present study examined the records of 20,422 separate hospitalisations involving polymedicated home-dwelling older patients, eventually discharged home, for the prevalence of 30-day hospital readmission. These records were held in four years of an exhaustive hospital register. The 13,802 individual older patients identified showed a 30-day hospital readmission rate of almost 8%, corroborating previously published all-cause hospital readmission rates among home-dwelling older patients (9, 27). However, Jencks *et al*. (2009) found a much higher 30-day readmission prevalence, reaching almost 20% among discharged older patients who had been hospitalised in acute medicine and surgery wards (3). As a bivariate association, multimorbid men were at a significantly higher risk of readmission than multimorbid women; however, in the adjusted multivariate analysis that significance disappeared. Medical conditions, PP and multiple classes of prescribed drugs were all associated with higher 30-day readmission rates, in line with previous studies (27, 52-54).

Our study found no significant differences in the risks of 30-day hospital readmission for men and women. However, some previous research found that men were more likely to forget to take their drugs or to not apply the changed drug dosages prescribed by their family physician, consequently increasing their risk of hospital readmission for drug-related problems (55). Opposite results were found in a population-based study by Manteufel *et al*.(56), with women being less likely than men to properly adhere to their drug prescriptions. These differences may indicate a need for more personalised drug prescription and drug management to improve clinical outcomes. Further research should explore associations between different types of drugs and sex (56, 57), but this topic was beyond the present

For peer review only - http://bmjopen.bmj.com/site/about/guidelines.xhtml

study's scope. Another interesting issue regarding sex differences in hospital readmission rates is the study window. Some studies found higher rates among men than among women below three-month readmissions. More extended time windows (e.g. one year) revealed no significant sex differences (52, 58). An analysis of our dataset using a more extended readmission window might clarify this point and provide complementary knowledge about sex-associated hospital readmissions.

Our results indicated that ageing was not a risk factor for increased 30-day hospital readmission, in line with some previous publications (53, 59). However, other research findings demonstrated that age was only positively associated with the likelihood of readmission up to 74 years old; above that, there no longer appeared to be any significant relationship between age and readmission (60, 61). These contrasting results may be explained by the studies' designs, country settings, the ages of their research populations or the medical conditions included (53, 60, 62).

Longer hospital stays were also associated with a higher risk of hospital readmission, in line with a cohort study by Sud *et al.* concluding that an extended hospital LOS was associated with increased rates of all types of readmission, except for hospitalisation after heart failure, where a short LOS was associated with increased rates of readmission for cardiovascular disease and heart failure (63).

Our results indicated a significant positive association between the number of a patient's medical conditions and the 30-day hospital readmission rate, confirming other recent retrospective hospital register studies (64, 65). More specifically, older patients with impaired mobility showed an increased risk of hospital readmission. This result was not surprising, bearing in mind that although these older patients were discharged home—and not to a nursing home—after their hospital stay, their health status might nevertheless require future readmission. Indeed, this corroborated publications about older patients discharged after orthopaedic treatment or who had been initially admitted for heart failure, myocardial infarction or pneumonia, but also presented with impaired mobility (66, 67).

Cognitive impairment was not associated with increased 30-day hospital readmission rates, in line with findings from the systematic review by Pickens *et al.*, which pointed out that dementia had a modest impact on readmission rates (68). It was no surprise that inpatients hospitalised for cancer faced a high risk of readmission, corroborating prior studies by Buhenn *et al.*, Chang *et al.* and Butcher (69-71).

PP significantly increased the 30-day hospital readmission rate, but this result was based on the average number of drugs prescribed to the sample of readmitted patients versus those not readmitted. Although PP was confirmed as a strong determinant of 30-day hospital readmission in publications by Leendertse *et al.* (72, 73), our results showed a progressive linear relationship between PP and readmission rate, and this should be interpreted with caution. Despite our results and other publications and research underlining the challenge of PP among multimorbid older patients, there is no overall consensus about the best way to deal with the broad general relationship between PP and hospital readmission (74).

Our advanced statistical analysis demonstrated that some specific drugs and the concomitant use of specific drug combinations were significantly associated with 30-day readmission risk, although this was not unexpected and has been confirmed in previous publications (37, 75). Mostly in line with the research findings of Zhang *et al.*, drugs including hormones, antineoplastics, immunosuppressors, neoplastic antibiotics and bacterial vaccines were substantial risk factors for hospital readmission (7).

For peer review only - http://bmjopen.bmj.com/site/about/guidelines.xhtml

In summary, extended hospital LOS, functional impairments, medical conditions and drugs have been demonstrated to be determinants of 30-day hospital readmission, although not all of them have clinically or pharmacologically relevant interpretations or explanations. Further research involving large samples is needed, notably to explore the drug–drug interactions with the highest risk of hospital readmissions. Statistical predictions of potential drug–drug interactions provide important information for modeling drug combinations and identifying pairs of drugs whose combination creates an exaggerated response (9). As the association between the number of drugs and the prevalence of hospital readmission was linear, more advanced inferential statistics would be needed to clarify a cut-off point for the mean number of drugs that would significantly increase the readmission rate. In addition, problems involving adherence to prescriptions, social support networks, and stronger or weaker primary health-care structures can all influence hospital readmission rates (39). According to some publications, nearly 70% of people aged over 65 make mistakes with their drugs (76, 77). Information about drug adherence, drug underuse and overuse, drug changes and deprescription by family physicians, as well as medication management at home would contribute to a more comprehensive understanding of disease- and drug-related 30-day hospital readmissions.

Strengths and Limitations

This study's main strength was its use of comprehensive and recorded data from an exhaustive register. We consider this retrospective study useful for clinical practice and future research because a whole series of sociodemographic and clinical parameters, medical conditions and prescribed drugs were used to predict the probability of hospital readmission. Using both bivariate and multivariate analyses enabled an evaluation of the data's longitudinal nature.

Our study had several limitations, nevertheless. The design did not allow us to identify hospitalisations and readmissions lost-to-follow-up and to adjust our data for death. We were also unable to identify unnecessary hospitalisations or any bias towards hospitalisation rather than another health-care solution among participants. Our dataset could not inform us about whether older inpatients had been first admitted to another hospital or were subsequently readmitted elsewhere during the study period. Another limitation was the study's lack of formal screening methods to explain ADEs in detail, and it was impossible to distinguish between elective and urgent hospitalisations. Although the study considered statistical associations between drugs and rehospitalisations, it did not use clinically diagnosed drug–drug interactions. Finally, we were unable to consider any potential causality between PP and hospital readmission.

Conclusions

Hospital length of stay, medical conditions, functional impairments and prescribed drugs were all critical factors in predicting hospital readmissions, thus affirming our hypotheses. Readmission patterns are complex and poorly understood because older patients often present with multiple chronic conditions, functional impairments and complex drug prescriptions. Hospital readmission is an under-investigated

For peer review only - http://bmjopen.bmj.com/site/about/guidelines.xhtml

topic deserving of additional, well-conducted, predictive research exploiting accurate longitudinal data from large samples.

Acknowledgments

The authors thank the partner hospital, including the hospital's data warehouse, for its valuable contributions. This research was developed, in part, using grants from the Swiss National Science Foundation and the School of Health Sciences of the University of Applied Sciences and Arts Western Switzerland (HES-SO) Valais/Wallis. The funders had no role in the design and conduct of the study, the collection, management, analysis and interpretation of the data, the preparation, review or approval of the manuscript, or the decision to submit the manuscript for publication.

Authors Contributions

All the authors contributed to data analysis or interpretation, to drafting or revising the article, gave final approval to the version to be published and agree to be accountable for all aspects of the work.

Funding

This study was supported by the Swiss National Science Foundation via grant number 407440_183434/1.

Competing interest

The authors report no conflicts of interest surrounding this work.

Ethics approval and patient consent

Ethical approval was obtained from the Human Research Ethics Committee of the Canton of Vaud (CER-VD, 2018-02196), thus permitting our partner hospital's data warehouse to provide the appropriate dataset. Given the retrospective data source, obtaining consent from the patients concerned was impossible or posed disproportionate difficulties. The present study respects the legal requirements for research projects involving data re-use without consent, as set out in Art. 34 from the Swiss Human Research Act (HTA).

Data sharing statement

As part of the Data Use Agreement, authors are not allowed to provide raw data. Upon a reasonable request, the corresponding author will provide statistical programming code used to generate results.

Word Count: 4,158

References

1. Rayan-Gharra N, Rn ES, Tadmor B, Flaks-Manov N, Balicer RD. Patients' ratings of the in-hospital discharge briefing and post-discharge primary care follow-up: the association with 30-day readmissions. Patient Education and Counseling. 2019.
2. Kabue S, Greene J, Kipnis P, Lawson B, Rinetti-Vargas G, Liu V, et al. The Impact of Pharmacy-specific Predictors on the Performance of 30-Day Readmission Risk Prediction Models. Med Care. 2019;57(4):295-9.
3. Jencks SF, Williams MV, Coleman EA. Rehospitalizations among Patients in the Medicare Fee-for-Service Program. New England Journal of Medicine. 2009;360(14):1418-28.

For peer review only

For peer review only - http://bmjopen.bmj.com/site/about/guidelines.xhtml

4. Covinsky KE, Palmer RM, Fortinsky RH, Counsell SR, Stewart AL, Kresevic DM. Loss of independence in activities of daily living in older adults hospitalised with medical illness: increased vulnerability with age. JAGS. 2003;51.

5. Hansen LO, Young RS, Hinami K, Leung A, Williams MV. Interventions to reduce 30-day rehospitalization: a systematic review. Ann Intern Med. 2011;155(8):520-8.

6. Davies EC, Green CF, Mottram DR, Rowe PH, Pirmohamed M. Emergency re-admissions to hospital due to adverse drug reactions within 1 year of the index admission. British Journal of Clinical Pharmacology. 2010;70(5):749-55.

7. Zhang M, Holman CDAJ, Price SD, Sanfilippo FM, Preen DB, Bulsara MK. Comorbidity and repeat admission to hospital for adverse drug reactions in older adults: retrospective cohort study. BMJ. 2009;338:a2752.

8. Bonnet-Zamponi D, d'Arailh L, Konrat C, Delpierre S, Lieberherr D, Lemaire A, et al. Drug-Related Readmissions to Medical Units of Older Adults Discharged from Acute Geriatric Units: Results of the Optimization of Medication in AGEd Multicenter Randomized Controlled Trial. Journal of the American Geriatrics Society. 2013;61(1):113-21.

9. Blanc A-L, Fumeaux T, Stirnemann J, Dupuis Lozeron E, Ourhamoune A, Desmeules J, et al. Development of a predictive score for potentially avoidable hospital readmissions for general internal medicine patients. PLOS ONE. 2019;14(7):e0219348.

10. Stevenson JM, Davies JG, Martin F, Ali K, Rajkumar C, Schiff R. Is medication related harm as a cause of readmission associated with the indicators of frailty? Age and Ageing. 2018;47:ii19.

11. Brunner-La Rocca H-P, Peden CJ, Soong J, Holman PA, Bogdanovskaya M, Barclay L. Reasons for readmission after hospital discharge in patients with chronic diseases—Information from an international dataset. PloS one. 2020;15(6):e0233457.

12. Dharmarajan K, Hsieh AF, Lin Z, Bueno H, Ross JS, Horwitz LI, et al. Hospital readmission performance and patterns of readmission: retrospective cohort study of Medicare admissions. Bmj. 2013;347.

13. Arora S, Patel P, Lahewala S, Patel N, Patel NJ, Thakore K, et al. Etiologies, trends, and predictors of 30-day readmission in patients with heart failure. The American journal of cardiology. 2017;119(5):760-9.

14. Shams I, Ajorlou S, Yang K. A predictive analytics approach to reducing 30-day avoidable readmissions among patients with heart failure, acute myocardial infarction, pneumonia, or COPD. Health care management science. 2015;18(1):19-34.

15. Krumholz HM, Wang K, Lin Z, Dharmarajan K, Horwitz LI, Ross JS, et al. Hospital-readmission risk—isolating hospital effects from patient effects. New England Journal of Medicine. 2017;377(11):1055-64.

16. Gruneir A, Fung K, Fischer HD, Bronskill SE, Panjwani D, Bell CM, et al. Care setting and 30-day hospital readmissions among older adults: a population-based cohort study. CMAJ. 2018;190(38):E1124-E33.

17. Marengoni A, Angleman S, Melis R, Mangialasche F, Karp A, Garmen A, et al. Aging with multimorbidity: A systematic review of the literature. Ageing Research Reviews. 2011;10:430-9.

18. Valderas JM, Starfield B, Sibbald B, Salisbury C, Rloand M. Defining comorbidity: implications for understanding health and health services. Annals Of Family Medicine. 2009;7:357-63.

19. Maher RL, Hanlon J, Hajjar ER. Clinical consequences of polypharmacy in elderly. Expert Opinion on Drug Safety. 2014;13:57-65.

20. Masnoon N, Shakib S, Kalisch-Ellett L, Caughey GE. What is polypharmacy? A systematic review of definitions. BMC Geriatrics. 2017;17:1-10.

21. Wastesson JW, Morin L, Tan ECK, Johnell K. An update on the clinical consequences of polypharmacy in older adults: a narrative review. Expert Opin Drug Saf. 2018;17(12):1185-96.

22. Rieckert A, Trampisch US, Klaaßen-Mielke R, Drewelow E, Esmail A, Johansson T, et al. Polypharmacy in older patients with chronic diseases: a cross-sectional analysis of factors associated with excessive polypharmacy. BMC Family Practice. 2018;19(1):113.

23. Magill SS, Edwards JR, Bamberg W, Beldavs ZG, Dumyati G, Kainer MA, et al. Multistate point-prevalence survey of health care-associated infections. N Engl J Med. 2014;370(13):1198-208.

24. Coleman EA, Smith JD, Raha D, Min SJ. Posthospital medication discrepancies: prevalence and contributing factors. Arch Intern Med. 2005;165(16):1842-7.

25. Ferreri SP, Hughes TD, Snyder ME. Medication Therapy Management: Current Challenges. Integr Pharm Res Pract. 2020;9:71-81.

For peer review only - http://bmjopen.bmj.com/site/about/guidelines.xhtml

26. Roux P, Verloo H, Santiago-Delefosse M, Pereira F. The spatial dimensions of medication management by home-dwelling older adults after hospital discharge. Health & Place. 2019;60:102230.
27. El Morabet N, Uitvlugt EB, van den Bemt BJF, van den Bemt P, Janssen MJA, Karapinar-Çarkit F. Prevalence and Preventability of Drug-Related Hospital Readmissions: A Systematic Review. J Am Geriatr Soc. 2018;66(3):602-8.
28. Forster AJ, Murff HJ, Peterson JF, Gandhi TK, Bates DW. The incidence and severity of adverse events affecting patients after discharge from the hospital. Ann Intern Med. 2003;138(3):161-7.
29. Yeo I, Cheung JW, Feldman DN, Amin N, Chae J, Wong SC, et al. Assessment of Hospital Readmission Rates, Risk Factors, and Causes After Cardiac Arrest: Analysis of the US Nationwide Readmissions Database. JAMA Network Open. 2019;2(9):e1912208-e.
30. Hauviller L, Eyvrard F, Garnault V, Rousseau V, Molinier L, Montastruc JL, et al. Hospital re-admission associated with adverse drug reactions in patients over the age of 65 years. European Journal of Clinical Pharmacology. 2016;72(5):631-9.
31. Davies EC, Green CF, Mottram DR, Rowe PH, M P. Emergency re-admissions to hospital due to adverse drug reactions within 1 year of the index admission. Br J Clin Pharmacol. 2010;70:749–55.
32. Samoy LJ, Zed PJ, Wilbur K, Balen RM, Abu-Laban RB, Roberts M. Drug-related hospitalizations in a tertiary care internal medicine service of a Canadian hospital: a prospective study. Pharmacotherapy. 2006;26(11):1578-86.
33. Teymoorian SS, Dutcher D, Woods M. ASSOCIATION BETWEEN POSTDISCHARGE ADVERSE DRUG REACTIONS AND 30-DAY HOSPITAL READMISSION IN PATIENTS AGED 80 AND OLDER. Journal of the American Geriatrics Society. 2011;59(5):948-9.
34. WHO. ADHERENCE TO LONG-TERM THERAPIES: Evidence for action. Geneva: World Health Organisation; 2003.
35. Brahma DK, Wahlang JB, Marak MD, Ch Sangma M. Adverse drug reactions in the elderly. J Pharmacol Pharmacother. 2013;4(2):91-4.
36. Davies EA, O'Mahony MS. Adverse drug reactions in special populations - the elderly. Br J Clin Pharmacol. 2015;80(4):796-807.
37. Moura CS, Tavares LS, Acurcio Fde A. [Hospital readmissions related to drug interactions: a retrospective study in a hospital setting]. Rev Saude Publica. 2012;46(6):1082-9.
38. Spinks JM, Kalisch Ellett LM, Spurling G, Theodoros T, Williamson D, Wheeler AJ. Adaptation of potentially preventable medication-related hospitalisation indicators for indigenous populations in Australia using a modified Delphi technique. BMJ Open. 2019;9(11):e031369.
39. Rosen OZ, Fridman R, Rosen BT, Shane R, Pevnick JM. Medication adherence as a predictor of 30-day hospital readmissions. Patient Prefer Adherence. 2017;11:801-10.
40. Pellegrin KL, Lee E, Uyeno R, Ayson C, Goo R. Potentially preventable medication-related hospitalizations: A clinical pharmacist approach to assessment, categorization, and quality improvement. Journal of the American Pharmacists Association. 2017;57(6):711-6.
41. Pereira F, Roux P, Santiago-Delefosse M, von Gunten A, Wernli B, Martins MM, et al. Optimising medication management for polymedicated home-dwelling older adults with multiple chronic conditions: a mixed-methods study protocol. BMJ Open. 2019;9(10):e030030.
42. Benchimol EI, Smeeth L, Guttmann A, Harron K, Moher D, Petersen I, et al. The REporting of studies Conducted using Observational Routinely-collected health Data (RECORD) Statement. PLOS Medicine. 2015;12(10):e1001885.
43. Strasberg HR, Geiger G, Tudiver F. Moving towards an electronic patient record: A survey to assess the needs of an academic family practice unit. Waegemann CP, editor. Newton: Medical Records Institute; 1998. A434-A43 p.
44. Surgical interventions clasification S. Swiss classification of surgical interventions (CHOP). 2016.
45. Parker S, Prince A, Thomas L, Song H, Milosevic D, Harris MF. Electronic, mobile and telehealth tools for vulnerable patients with chronic disease: a systematic review and realist synthesis. BMJ Open. 2018;8(8).
46. Olsen J. Register-based research: some methodological considerations. Scandinavian journal of public health. 2011;39(3):225-9.
47. Taushanov Z, Verloo H, Wernli B, Di Giovanni S, von Gunten A, F P. Transforming a patient registry into a customised dataset for the advanced statistical analysis of health risk factors and for medication-related hospitalisation research: a retrospective hospital patient registry study. JMIR Medical Informatics. 2021.
48. Andrade C. Age as a variable: Continuous or categorical? Indian J Psychiatry. 2017;59(4):524-5.

For peer review only

49. WHO. The Anatomical Therapeutic Chemical Classification System with Defined Daily Doses (ATC/DDD) Geneva: World Health Organization; 2014 [24 March 2020]. Available from: http://www.who.int/classifications/atcddd/en/.
50. Goldstein H. Nonlinear Multilevel Models, with an Application to Discrete Response Data. Biometrika. 1991;78(1):45-51.
51. Haider SI, Johnell K, Thorslund M, Fastbom J. Trends in polypharmacy and potential drug-drug interactions across educational groups in elderly patients in Sweden for the period 1992 - 2002. Int J Clin Pharmacol Ther. 2007;45(12):643-53.
52. Linkens AEMJH, Milosevic V, van der Kuy PHM, Damen-Hendriks VH, Mestres Gonzalvo C, Hurkens KPGM. Medication-related hospital admissions and readmissions in older patients: an overview of literature. International Journal of Clinical Pharmacy. 2020.
53. Berry JG, Gay JC, Joynt Maddox K, Coleman EA, Bucholz EM, O'Neill MR, et al. Age trends in 30 day hospital readmissions: US national retrospective analysis. BMJ. 2018;360:k497.
54. Kongkaew C, Hann M, Mandal J, Williams SD, Metcalfe D, Noyce PR, et al. Risk factors for hospital admissions associated with adverse drug events. Pharmacotherapy. 2013;33(8):827-37.
55. Thunander Sundbom L, Bingefors K. Women and men report different behaviours in, and reasons for medication non-adherence: a nationwide Swedish survey. Pharmacy practice. 2012;10(4):207-21.
56. Manteuffel M, Williams S, Chen W, Verbrugge RR, Pittman DG, Steinkellner A. Influence of patient sex and gender on medication use, adherence, and prescribing alignment with guidelines. J Womens Health (Larchmt). 2014;23(2):112-9.
57. Thunander Sundbom L, Hedborg K. Association between prescribed antidepressants and other prescribed drugs differ by gender: a nationwide register-based study in Sweden. Nord J Psychiatry. 2019;73(1):73-9.
58. Silverstein MD, Qin H, Mercer SQ, Fong J, Haydar Z. Risk Factors for 30-Day Hospital Readmission in Patients ≥65 Years of Age. Baylor University Medical Center Proceedings. 2008;21(4):363-72.
59. Schwab C, Hindlet P, Sabatier B, Fernandez C, Korb-Savoldelli V. Risk scores identifying elderly inpatients at risk of 30-day unplanned readmission and accident and emergency department visit: a systematic review. BMJ Open. 2019;9(7):e028302.
60. Jain S, Khera R, Mortensen EM, Weissler JC. Readmissions of adults within three age groups following hospitalization for pneumonia: Analysis from the Nationwide Readmissions Database. PLOS ONE. 2018;13(9):e0203375.
61. Hasegawa K, Gibo K, Tsugawa Y, Shimada YJ, Camargo CA, Jr. Age-Related Differences in the Rate, Timing, and Diagnosis of 30-Day Readmissions in Hospitalized Adults With Asthma Exacerbation. Chest. 2016;149(4):1021-9.
62. Shebeshi DS, Dolja-Gore X, Byles J. Unplanned Readmission within 28 Days of Hospital Discharge in a Longitudinal Population-Based Cohort of Older Australian Women. International Journal of Environmental Research and Public Health. 2020;17(9):3136.
63. Sud M, Yu B, Wijeysundera HC, Austin PC, Ko DT, Braga J, et al. Associations Between Short or Long Length of Stay and 30-Day Readmission and Mortality in Hospitalized Patients With Heart Failure. JACC: Heart Failure. 2017;5(8):578-88.
64. Donzé J, Lipsitz S, Bates DW, Schnipper JL. Causes and patterns of readmissions in patients with common comorbidities: retrospective cohort study. Bmj. 2013;347:f7171.
65. Hijazi HH, Alyahya MS, Hammouri HM, Alshraideh HA. Risk assessment of comorbidities on 30-day avoidable hospital readmissions among internal medicine patients. J Eval Clin Pract. 2017;23(2):391-401.
66. Falvey JR, Bade MJ, Hogan C, Forster JE, Stevens-Lapsley JE. Preoperative Activities of Daily Living Dependency is Associated With Higher 30-Day Readmission Risk for Older Adults After Total Joint Arthroplasty. Clin Orthop Relat Res. 2020;478(2):231-7.
67. Greysen SR, Stijacic Cenzer I, Auerbach AD, Covinsky KE. Functional impairment and hospital readmission in Medicare seniors. JAMA Intern Med. 2015;175(4):559-65.
68. Pickens S, Naik AD, Catic A, Kunik ME. Dementia and Hospital Readmission Rates: A Systematic Review. Dementia and Geriatric Cognitive Disorders Extra. 2017;7(3):346-53.
69. Chiang LY, Liu J, Flood KL, Carroll MB, Piccirillo JF, Stark S, et al. Geriatric assessment as predictors of hospital readmission in older adults with cancer. J Geriatr Oncol. 2015;6(4):254-61.
70. Butcher L. Oncologists Seek to Understand, Address Hospital Readmissions. Oncology Times. 2016;38(6):1,9-10.

For peer review only

For peer review only - http://bmjopen.bmj.com/site/about/guidelines.xhtml

71. Burhenn P, Sun C-L, Scher KS, Hsu J, Pandya P, Chui C-Y, et al. Predictors of hospital readmission among older adults with cancer. J Geriatr Oncol. 2020;11(7):1108-14.
72. Leendertse AJ, Egberts ACG, Stoker LJ, van den Bemt PMLA, Group HS. Frequency of and Risk Factors for Preventable Medication-Related Hospital Admissions in the Netherlands. Archives of Internal Medicine. 2008;168(17):1890-6.
73. Leendertse AJ, Van Den Bemt PM, Poolman JB, Stoker LJ, Egberts AC, Postma MJ. Preventable hospital admissions related to medication (HARM): cost analysis of the HARM study. Value Health. 2011;14(1):34-40.
74. Garfinkel D, Bilek A. Inappropriate medication use and polypharmacy in older people. BMJ. 2020;369:m2023.
75. Tesfaye WH, Peterson GM, Castelino RL, McKercher C, Jose MD, Wimmer BC, et al. Medication Regimen Complexity and Hospital Readmission in Older Adults With Chronic Kidney Disease. Annals of Pharmacotherapy. 2019;53(1):28-34.
76. Fialová D, Onder G. Medication errors in elderly people: contributing factors and future perspectives. British journal of clinical pharmacology. 2009;67(6):641-5.
77. Lavan AH, Gallagher PF, O'Mahony D. Methods to reduce prescribing errors in elderly patients with multimorbidity. Clinical interventions in aging. 2016;11:857-66.

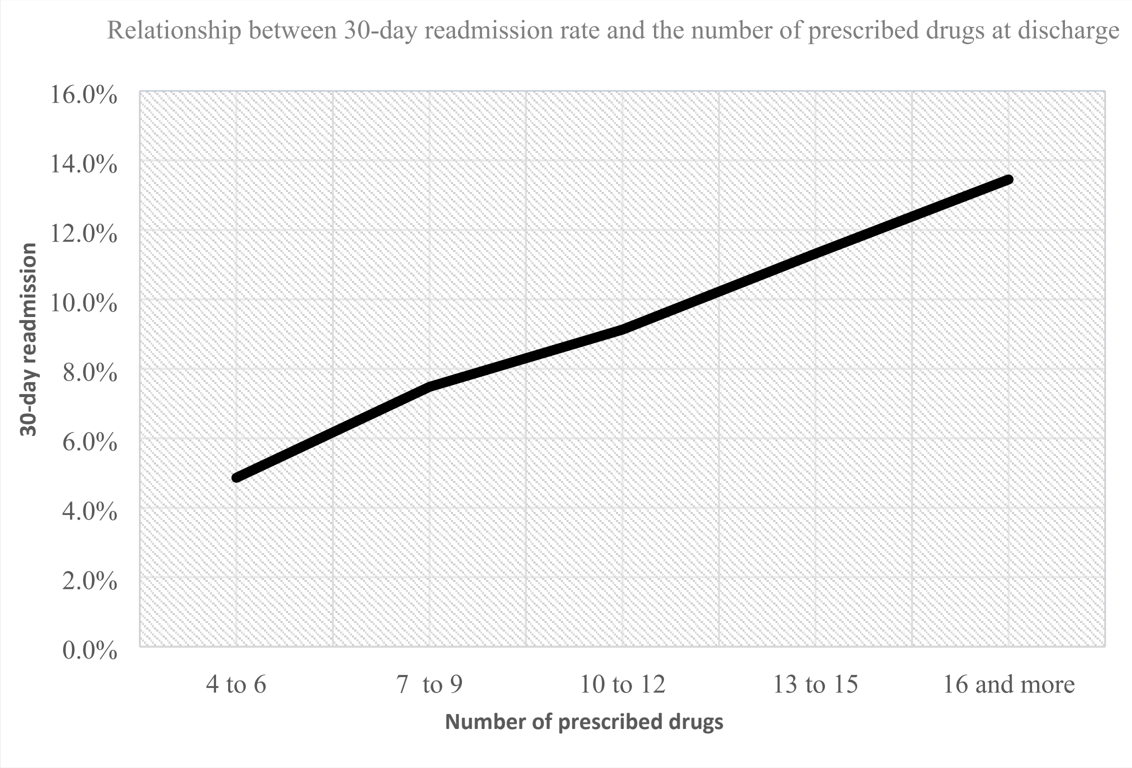

Figure 1. Relationship between 30-day readmission rate and the number of prescribed drugs at discharge.

Table 1. Descriptive statistics of drugs prescribed per patient (N = 13,802) at discharge based on the ATC Classification System.

**Drug classes based on the ATC Classification System**	**Min-Max**	**Mean (SD)**
First level, anatomical main group		
Blood and blood forming organs (B)	0–5	1.15 (0.86)
Dermatologicals (D)	0–3	0.04 (0.21)
Genitourinary system and sex hormones (G)	0–4	0.21 (0.47)
Systemic hormonal preparations, excl. sex hormones and insulins (H)	0–4	0.20 (0.46)

For peer review only - http://bmjopen.bmj.com/site/about/guidelines.xhtml

Anti-infectives for systemic use (J)	0–4	0.24 (0.47)
Antineoplastic and immunomodulating agents (L)	0–5	0.05 (0.23)
Musculo-skeletal system (M)	0–3	0.15 (0.39)
Antiparasitic products, insecticides and repellents (P)	0–2	0.02 (0.13)
Respiratory system (R)	0–7	0.28 (0.72)
Sensory organs (S)	0–6	0.10 (0.39)
Second level, therapeutic subgroup		
Stomatological preparations (A01)	0–1	0.00 (0.06)
Drugs for acid-related disorders (A02)	0–3	0.56 (0.52)
Drugs for functional gastrointestinal disorders (A03)	0–3	0.07 (0.28)
Antiemetics and antinauseants (A04)	0–1	0.01 (0.08)
Bile and liver therapy (A05)	0–1	0.00 (0.05)
Drugs for constipation (A06)	0–3	0.15 (0.40)
Antidiarrhoeals, intestinal anti-inflammatory/anti-infective agents (A07)	0–2	0.03 (0.18)
Digestives, incl. Enzymes (A09)	0–2	0.02 (0.13)
Drugs used in diabetes (A10)	0–5	0.26 (0.63)
Vitamins (A11)	0–4	0.15 (0.44)
Mineral supplements (A12)	0–3	0.29 (0.51)
Other alimentary tract and metabolism products (A16)	0–1	0.00 (0.05)
Cardiac therapy drugs (C01)	0–4	0.14 (0.42)
Antihypertensives (C02)	0–2	0.02 (0.17)
Diuretics (C03)	0–3	0.27 (0.53)
Peripheral vasodilators (C04)	0–1	0.00 (0.06)
Vasoprotectives (C05)	0–3	0.02 (0.14)
Beta-blocking agents (C07)	0–2	0.46 (0.51)
Calcium channel blockers (C08)	0–2	0.16 (0.37)
Agents acting on the renin-angiotensin system (C09)	0–3	0.64 (0.62)
Lipid modifying agents (C10)	0–3	0.43 (0.52)
Anaesthetics (N01)	0–1	0.00 (0.05)
Analgaesics (N02)	0–7	1.02 (0.91)
Antiepileptics (N03)	0–5	0.11 (0.35)
Drugs for Parkinson's disease (N04)	0–5	0.04 (0.24)
Psycholeptics (N05)	0–6	0.53 (0.73)
Psychoanaleptics (N06)	0–3	0.20 (0.44)
Other nervous system drugs(N07)	0–3	0.03 (0.19)
**Total number of drugs**	**5–30**	**8.95 (3.24)**

Table 2. Prevalence of hospital 30-day readmissions at different periods for different age groups (N = 13,802).

Variables	30-day hospital readmission	*p*-value
Complete sample	7.8%	
Sex		*
Women vs men	7.3% vs 8.2%	
Year-end age, in years		NS
65–69<br>70–79<br>80–89<br>≥ 90	7.5%<br>7.6%<br>8.4%<br>6.4%	
Mobility cluster:		NS
Preserved mobility vs impaired mobility	7.6% vs 8.5%	

For peer review only - http://bmjopen.bmj.com/site/about/guidelines.xhtml

Activities in daily living (ADL):		NS
Full ADL ability vs impaired ADL	7.8% vs 7.2%	
Cognitive status:		NS
Preserved cognitive status vs cognitive impairment	7.8% vs 7.9%	
ICD-10 diagnosis: circulatory problems		**
No vs Yes	8.2% vs 6.5%	
ICD-10 diagnosis: infection		NS
No vs Yes	7.7% vs 9.9%	
ICD-10 diagnosis: respiratory problems		NS
No vs Yes	7.8% vs 8.0%	
ICD-10 diagnosis: trauma		**
No vs Yes	8.0% vs 5.8%	
ICD-10 diagnosis: tumour		***
No vs Yes	6.9% vs 15.1%	
Number of ICD-10 conditions		***
1	1.5%	
2	4.9%	
3	3.6%	
4	4.8%	
5	8.8%	
Number of surgical procedures (CHOP)		*
0	7.7%	
1	7.8%	
2	7.0%	
3	7.3%	
4	7.1%	
5	9.7%	
Year: 2015–2018		NS
2015	8.3%	
2016	8.0%	
2017	8.0%	
2018	6.8%	

Note. * = $p < 0.05$; ** = $p < 0.01$; *** = $p < 0.001$; NS = non-significant

Table 3. Prevalence of readmission for different classes of drugs based on the ATC (N = 13,802).

**Drug class**	**30-day readmission with NO drugs in this class**	**30-day readmission with drugs in this class**	***p*-value**
First level, anatomical main group			
Blood and blood-forming organ drugs (B)	7.1%	8.0%	NS
Dermatologicals (D)	7.7%	9.4%	NS
Genitourinary system and sex hormones (G)	7.7%	8.3%	NS
Systemic hormonal preparations, excluding sex hormones and insulins (H)	7.4%	9.5%	***
Anti-infectives for systemic use (J)	8.0%	7.2%	NS
Antineoplastic and immunomodulating agents (L)	7.6%	12.6%	***
Drugs for the musculo-skeletal system (M)	8.0%	6.5%	*
Antiparasitic products, insecticides, and repellents (P)	7.8%	6.6%	***
Respiratory system drugs (R)	7.4%	9.9%	***
Sensory organ drugs (S)	7.8%	8.4%	NS

For peer review only - http://bmjopen.bmj.com/site/about/guidelines.xhtml

Second level, therapeutic subgroup			
Stomatological preparations (A01)	7.8%	12.2%	NS
Drugs for acid-related disorders (A02)	7.0%	8.5%	***
Drugs for functional gastrointestinal disorders (A03)	7.4%	13.4%	***
Antiemetics and antinauseants (A04)	7.7%	27.7%	***
Bile and liver therapy drugs (A05)	7.8%	14.3%	NS
Drugs for constipation (A06)	7.3%	10.8%	***
Antidiarrhoeals, intestinal anti-inflammatory/anti-infective agents (A07)	7.7%	12.9%	***
Digestives, including enzymes (A09)	7.8%	10.0%	NS
Drugs used in diabetes (A10)	7.4%	9.5%	***
Vitamins (A11)	7.5%	9.9%	***
Mineral supplements (A12)	7.4%	8.8%	**
Other alimentary tract and metabolism products (A16)	7.8%	6.3%	NS
Cardiac therapy (C01)	7.6%	8.9%	NS
Antihypertensives (C02)	7.7%	14.1%	***
Diuretics (C03)	7.2%	9.8%	***
Peripheral vasodilators (C04)	7.8%	15.2%	NS
Vasoprotective drugs (C05)	7.8%	9.8%	NS
Beta-blocking agents (C07)	7.1%	8.6%	***
Calcium channel blockers (C08)	7.7%	8.6%	NS
Agents acting on the renin-angiotensin system (C09)	8.7%	7.1%	***
Lipid-modifying agents (C10)	8.3%	7.1%	**
Anaesthetics (N01)	7.8%	18.8%	*
Analgaesics (N02)	7.8%	7.8%	NS
Antiepileptics (N03)	7.7%	9.0%	NS
Drugs for Parkinson's disease (N04)	7.8%	6.9%	NS
Psycholeptics (N05)	6.8%	9.3%	***
Psychoanaleptics (N06)	7.8%	7.7%	NS
Other nervous system drugs (N07)	7.9%	5.1%	NS

Note. * = $p < 0.05$; ** = $p < 0.01$; *** = $p < 0.001$; NS = non-significant

Table 4. Baseline, multilevel, logistic regression model using 30-day readmission (0 = no; 1 = yes) as the dependent variable associated with independent sociodemographic, LOS, and clinical variables (N = 13,802 observations for 8,878 different participants).

**Variables**	**Odds Ratio**[3]	***P* > z**	**95% CI** [4]
Sex	1.079	0.285	0.938–1.242
Year-end age, in years	0.999	0.878	0.990–1.009
Hospital length of stay (LOS), in days	1.014	0.000	1.006–1.021
Mobility cluster [1]	1.218	0.015	1.039–1.427
Dependency in the activities of daily living[1]	0.794	0.248	0.537–1.174
Mental health status[1]	0.992	0.966	0.687–1.433
CIM 1 diagnosis: circulatory problems [2]	0.938	0.491	0.783–1.124
CIM 1 diagnosis: infection [2]	1.381	0.078	0.964–1.977
CIM 1 diagnosis: respiratory problems [2]	1.100	0.414	0.875–1.382
CIM 1 diagnosis: trauma [2]	0.847	0.265	0.633–1.134
CIM 1 diagnosis: tumour [2]	2.538	0.000	2.089–3.082
Number of CIM	1.419	0.000	1.282–1.572

For peer review only - http://bmjopen.bmj.com/site/about/guidelines.xhtml

For peer review only

Number of CHOP	0.978	0.304	0.938–1.020
Number of drugs	1.043	0.000	1.028–1.058
Year: 2015 to 2018	0.933	0.022	0.880–0.990
Intercept	.	0.027	.

Note. 1: 0 = good state, 1 = impairment; 2: 0 = no, 1 = yes; 3: adjusted Odds ratio; 4:

Table 5. Multilevel logistic regression model results for the drugs prescribed to older patients at discharge home that had significant predictive values (odds ratios) for 30-day hospital readmission (controlled for variables in the baseline model: Table 4) (N = 13,802 observations for 8,878 different participants).

**Variables**	**Odds ratio**[1]	***p* > z**	**95% CI** [2]
First level, anatomical main group			
Blood and blood-forming organs drugs (B)	1.089	0.041	1.003–1.181
Systemic hormonal preparations, excluding sex hormones and insulins (H)	1.207	0.007	1.052–1.385
Respiratory system drugs (R)	1.146	0.003	1.046–1.254
Second level, therapeutic subgroup			
Drugs for functional gastrointestinal disorders (A03)	1.424	0.001	1.166–1.739
Antiemetics and antinauseants (A04)	3.216	0.000	1.842–5.617
Drugs for constipation (A06)	1.195	0.018	1.031–1.386
Drugs used in diabetes (A10)	1.125	0.021	1.018–1.243
Vitamins (A11)	1.201	0.008	1.049–1.374
Antihypertensives (C02)	1.771	0.000	1.287–2.438
Diuretics (C03)	1.149	0.024	1.018–1.296
Beta-blocking agents (C07)	1.156	0.040	1.007–1.327
Lipid-modifying agents (C10)	0.841	0.015	0.732–0.967
Psycholeptics (N05)	1.130	0.009	1.031–1.238

Note. 1 = adjusted odds ratio; 2 = CI or Confidence Interval

Table 6. Drugs and drugs interactions from ATC classes A and B with a significant risk of 30-day hospital readmission (controlled for variables in the baseline model: Table 4) (N = 13,802 observations for 8,878 different participants).

**Variables**	**Odds ratio**[1]	***p* > z**	**95% CI** [2]
First level, anatomical main group			
Blood and blood-forming organ drugs (B)	1.089	0.040	1.004–1.182
Systemic hormonal preparations, excluding sex hormones and insulins (H)	1.210	0.007	1.054–1.390
Respiratory system drugs (R)	1.149	0.003	1.049–1.258
Second level, therapeutic subgroup			
Antiemetics and antinauseants (A04)	3.222	0.000	1.844–5.630

For peer review only - http://bmjopen.bmj.com/site/about/guidelines.xhtml

For peer review only

Drugs for functional gastrointestinal disorders (A03)	1.428	0.000	1.169–1744
Beta-blocking agents (C07) and drugs for acid-related disorders (A02)	1.367	0.022	1.046–1.788
Drugs for constipation (A06)	1.199	0.017	1.033–1.392
Agents acting on the renin-angiotensin system (C09)	0.892	0.049	0.796–0.999
Lipid-modifying agents (C10)	0.838	0.013	0.729–0.964

Note. 1: adjusted odds ratio; 2: CI = Confidence Interval

For peer review only

For peer review only - http://bmjopen.bmj.com/site/about/guidelines.xhtml

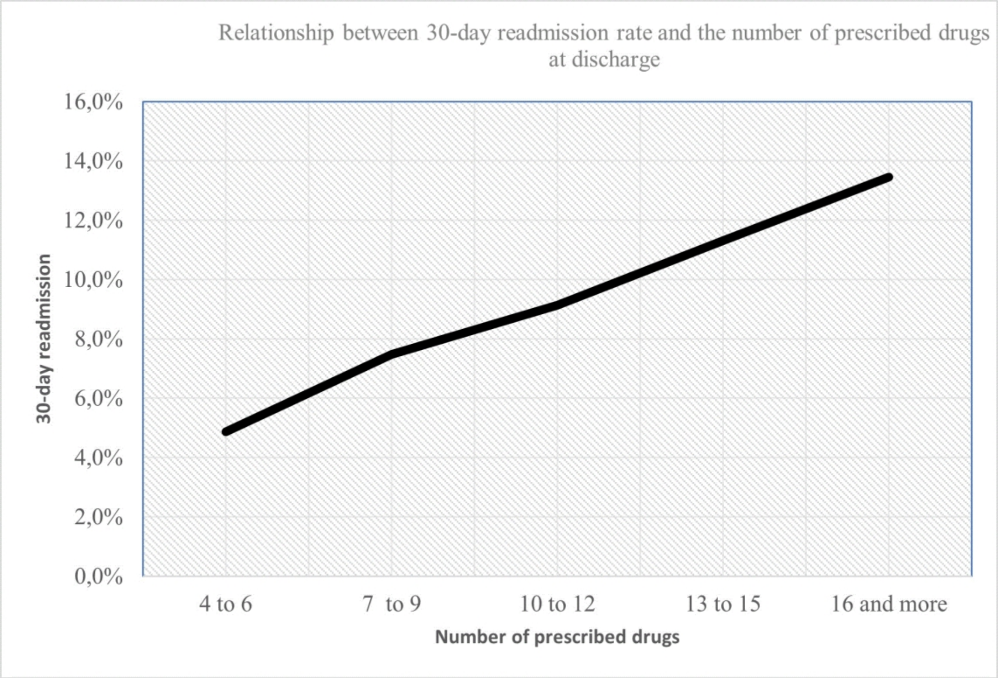

Figure 1. Relationship between 30-day readmission rate and the number of prescribed drugs at discharge.

688x467mm (72 x 72 DPI)

Open access Protocol

BMJ Open

Optimising medication management for polymedicated home-dwelling older adults with multiple chronic conditions: a mixed-methods study protocol

Filipa Pereira [1,2] Pauline Roux,[3] Marie Santiago-Delefosse,[3] Armin von Gunten,[4] Boris Wernli,[5] Maria Manuela Martins,[2,6] Henk Verloo[1,4]

To cite: Pereira F, Roux P, Santiago-Delefosse M, *et al*. Optimising medication management for polymedicated home-dwelling older adults with multiple chronic conditions: a mixed-methods study protocol. *BMJ Open* 2019;**9**:e030030. doi:10.1136/bmjopen-2019-030030

► Prepublication history and additional material for this paper are available online. To view these files, please visit the journal online (http://dx.doi.org/10.1136/bmjopen-2019-030030).

Received 23 February 2019
Revised 17 August 2019
Accepted 03 October 2019

Check for updates

© Author(s) (or their employer(s)) 2019. Re-use permitted under CC BY-NC. No commercial re-use. See rights and permissions. Published by BMJ.

For numbered affiliations see end of article.

Correspondence to
Filipa Pereira;
filipa.pereira@hevs.ch

ABSTRACT

Introduction Optimal medication management is one of the basic conditions necessary for home-dwelling older adults living with multiple chronic conditions (OAMCC) to be able to remain at home and preserve their quality of life. Currently, the reasons for such high numbers of emergency department visits and the very significant rate of hospitalisations for OAMCC, due to medication-related problems (MRPs), is poorly explored. This study aims to reveal the current state of the medication management practices of polymedicated, home-dwelling OAMCC and to make proposals for improving clinical and medication pathways through an innovative and integrated model for supporting medication management and preventing adverse health outcomes.

Methods and analysis A mixed-methods study will address the medication management of polymedicated, home-dwelling OAMCC. Its explanatory sequential design will involve two major phases conducted sequentially over time. The quantitative phase will consist of retrospectively exploiting the last 5 years of electronic patient records from a local hospital (N ≈ 50 000) in order to identify the different profiles—made up of patient-related, medication-related and environment-related factors—of the polymedicated, home-dwelling OAMCC at risk of hospitalisation, emergency department visits, hospital readmission (notably for MRPs), institutionalisation or early death. The qualitative study will involve: (a) obtaining and understanding the medication management practices and experiences of the identified profiles extracted from the hospital data of OAMCC who will be interviewed at home (N ≈ 30); (b) collecting and analysing the perspectives of the formal and informal caregivers involved in medication management at home in order to cross-reference perspectives about this important dimension of care at home. Finally, the mixed-methods findings will enable the development of an innovative, integrated model of medication management based on the Agency for Clinical Innovation framework and Bodenheimer and Sinsky's quadruple aim.

Ethics and dissemination Ethical approval has been obtained from the Human Research Ethics Committee of the Canton Vaud (2018-02196). Findings will be disseminated in peer-reviewed journals, professional conferences and other knowledge transfer activities with primary healthcare providers, hospital care units, informal caregivers' and patients' associations.

Strengths and limitations of this study

- ► This mixed methodology will rely on a closely coordinated combination of methods and on the utilisation of valuable existing data underexploited to date (patients' electronic hospital records and Resident Assessment Instrument-Home Care (RAI-HC) data).
- ► The investigation draws on an interprofessional and interdisciplinary approach, which associates general practitioners, community healthcare nurses, pharmacists and researchers in health psychology, old age psychiatry, nursing and survey methodology.
- ► Our findings will contribute to the development of an evidence-based and innovative, cooperative model of medication management for polymedicated, home-dwelling older adults with multiple chronic conditions.
- ► Although patients' electronic hospital records and RAI-HC data provide a broad range of patient-related, medication-related and environment-related information, they rarely highlight factors that may influence the occurrence of medication-related problems.

INTRODUCTION

The number of older adults living at home with multiple chronic conditions (OAMCC) rises considerably around the world and has been estimated to affect 25.2% of people aged from 65 to 79% and 41.3% of those aged 80 and over.[1] Multiple chronic conditions is a comprehensive concept used to properly cover the diverse definitions of multimorbidity[2 3] and therefore the complexity of older adults' health statuses. The concept encompasses the simultaneous presence of an individual's diseases and their chronic physical, mental or behavioural health problems requiring ongoing management over years or decades.[4]

These long-term health conditions require taking multiple medications,[5] known as polypharmacy (PP) when the daily intake

For peer review only - http://bmjopen.bmj.com/site/about/guidelines.xhtml

BMJ Open: first published as 10.1136/bmjopen-2019-030030 on 28 October 2019. Downloaded from http://bmjopen.bmj.com/ on November 12, 2019 at HES-SO Valais-Wallis - Fil?re Soins Infirmiers. Protected by copyright.

corresponds to five or more medicines.[6] PP places older adults at higher risk of medication-related problems (MRPs), including adverse medication reactions, medication errors and potentially inappropriate medications.[7 8] Potentially inappropriate medications are the intake of medicines for which the associated risks outweigh the potential benefits, particularly when more effective alternatives are available.[9] Consequently, MRPs can lead to a degradation of the patient's clinical condition, physical and cognitive decline, an exacerbation of chronic medical conditions and avoidable health costs.[10 11] Moreover, up to 25% of emergency department visits by home-dwelling OAMCC are due to MRPs.[10] However, 60% of MRPs in patients visiting the emergency department with non-specific complaints (such as weakness) may go undiagnosed, whereas 83% of those MRPs may be responsible for acute morbidity.[10] MRPs are also a frequent cause of readmission, and they were the most frequent cause in one study that followed older patients for 6 months after hospital discharge.[12] Care-coordination problems, associated with low or suboptimal medication management, are all the more evident in the sensitive period of discharge home from hospital.[11 13] The complexity of OAMCC's care needs leads them to be significant users of health services and to consult many different healthcare professionals.[14] The number of healthcare professionals consulted by home-dwelling OAMCC has been directly associated with fragmented and uncoordinated care.[13] Moreover, different healthcare professionals may have different treatment preferences. Failure to coordinate care among home-dwelling OAMCC contributes to MRPs.[13]

In addition to the role of healthcare professionals in medication management, informal caregivers play a vital role in ensuring safe and appropriate medication use by home-dwelling OAMCC, especially among those who may also have cognitive impairment.[15–17] Despite the important role of informal caregivers in medication management, several complications to do with their activities have been documented in relation to the time spent, anxiety making a mistake and the uncooperative behaviour of the home-dwelling OAMCC.[18] They are also confronted with difficulties in maintaining continuous supplies of medication, assisting with administration, making clinical judgements (eg, in response to side effects and about over-the-counter medication), and solving conflictual communications or disagreements with the older adult,[18] or even with healthcare professionals, with regard to ineffective and addictive medication practices.[15 18]

Nonetheless, many MRPs are preventable.[8 10 19] Studies about medicine-related hospitalisations suggest that up to 58% may be preventable with appropriate primary care.[8] An essential strategy for medicine-related hospitalisations prevention and medication safety is medication reconciliation—the process of creating and maintaining a single list of the patient's current list of medications.[20] This process allows a systematic and comprehensive review of all the medications the patient is taking, reducing medication errors by a consistent communication across transitions of care.[21]

Therefore, optimising medication management among home-dwelling OAMCC requires regular monitoring of MRPs, interprofessional collaboration across different health and social care providers, organisations and departments[13] and medication reconciliation at every transition of care including changes in the clinical setting, practitioner or level of care.[22]

Aim and objectives

This study's aim is to document the current state of medication management practices of polymedicated, home-dwelling OAMCC and to make proposals for improving evidence-based clinical and medication pathways through an innovative, integrated model intended to support medication management and to prevent adverse health outcomes related to MRPs (recurrent hospitalisation, emergency department visits, institutionalisation in nursing homes and early death). To achieve this aim, three main objectives will guide this project:

The first objective is to carry out a *retrospective analysis of patients'* hospital records, their medication and environment-related factors in order to identify those that increase the risk of hospitalisation, emergency department visits, hospital readmission (notably due to MRPs), institutionalisation or early death, among home-dwelling polymedicated OAMCC—factors that prevent OAMCC from staying at home.

The second objective is to use *a prospective qualitative study* to explore and better understand *the medication experiences and practices of home-dwelling OAMCC with different profiles*. We seek to identify the skills and strategies developed by them to manage polymedication within their social contexts and health trajectories despite possible cognitive impairment and particularly after a recent hospitalisation.

The third objective is to better understand the *roles and coordination of the different caregivers involved in the medication management of home-dwelling OAMCC*. We seek to investigate the perspectives of both professional caregivers (community healthcare nurses, pharmacists, general practitioners or specialist physicians) and non-professional/informal caregivers (family members, friends or neighbours).

METHODS

Study design

To enable us to meet our objectives, a mixed-methods study will address the medication management of polymedicated, home-dwelling OAMCC.[23] Two major phases will be conducted sequentially from February 2019 to January 2022: a quantitative data collection phase followed by a qualitative phase. The reasons for using an explanatory sequential design are, first, that existing data in electronic patient records from a local hospital will enable us to identify profiles affected by similar

For peer review only - http://bmjopen.bmj.com/site/about/guidelines.xhtml

BMJ Open: first published as 10.1136/bmjopen-2019-030030 on 28 October 2019. Downloaded from http://bmjopen.bmj.com/ on November 12, 2019 at HES-SO Valais-Wallis - Fil?re Soins Infirmiers. Protected by copyright.

patient-related, medication-related and environment-related factors among the polymedicated, home-dwelling OAMCC at risk of hospitalisation, emergency department visits, hospital readmission (notably due to MRPs), institutionalisation or early death. Second, the identified profiles extracted from the hospital data will allow proceeding to a purposive sampling—of those polymedicated, home-dwelling OAMCC who present with more risk factors—for the qualitative data collection focused on medication management at home. Thus, the analysis of the results from the retrospective quantitative phase will be integrated with the data collected from the prospective qualitative phase. Finally, phase 3 will develop a Medication Management Model based on interpreting the quantitative and qualitative findings.

Phase 1: retrospective quantitative analysis

To fulfil the first objective, the purpose of the quantitative phase is to identify the different profiles—made up of patient-related, medication-related and environment-related factors—of the polymedicated, home-dwelling OAMCC at risk of hospitalisation, emergency department visits, hospital readmission (notably for MRPs), institutionalisation in nursing homes or early death (before the average age of death described by the Organisation for Economic Cooperation and Development in 2018).[24] A systematic, retrospective chart analysis of the electronic patient records from a local hospital over the last 4 years using the evidence-based methodology developed by Vassar and Holzmann will provide substantial clinical information.[25] Motheral *et al*'s standardised extraction sheets will be adapted to explore and assess the data of older inpatients or emergency department-visiting home-dwelling older adults.[26] The 4-year analysis was selected based on the availability of systematic, well-coded patient data using the Swiss-Diagnostic Related Groups[27] and the Swiss Classification of Surgical Interventions (CHOP).[28]

Research population

All home-dwelling OAMCC with somatic and/or mental health disorders who were hospitalised, rehospitalised or who consulted the emergency department (for MRPs or other reasons) at the partner hospital between 2015 and 2018 (estimated n=50 000) will be included. The estimated sample of 50 000 older adults' electronic inpatient charts are part of the 40 000 yearly adult inpatients in acute care units and more than 40 000 adult emergency department consultations yearly at the partner hospital. To explore generalisability, we will compare their sociodemographic and health status characteristics with those of the national sample of hospitalised older adults in Swiss hospitals for the same period.

Data collection

Data from the hospitalisation and emergency admissions databases will be collected on patient-related, medication-related and environment-related factors that could have influenced the occurrence of MRPs that resulted in hospitalisation, rehospitalisation or emergency department admission.

Patient-related factors comprise sociodemographic characteristics, the International Classification of Diseases 10th version (ICD-10) diagnostics (main diagnosis and comorbidities), the Swiss Classification of Surgical Interventions (CHOP) category and the reason for hospitalisation, rehospitalisation or emergency department admission. Supplementary filters will be added to discriminate polymedication, multimorbidity (secondary ICD-10 diagnosis), physical and cognitive impairment documented in the clinical data files (Function Independence Measure, Mini-Mental State Examination and Activities of Daily Living).

Medication-related factors include the number, types and changes in medication at admission, during hospitalisation and at discharge.

Environment-related factors include the presence of formal and/or informal caregivers, patient's provenance (rural or urban), hospital pathways (wards and eventual transfers), length of stay, readmissions (number of admissions in the previous year, 30-day readmission and unplanned readmission), discharge destination and, potentially, death during hospitalisation. A unique patient identification number will allow us to analyse rehospitalisations via the emergency department during the period from 2015 to 2018. Retrospective data collection began in April 2019.

Data analyses

The data set of polymedicated, home-dwelling OAMCC will be analysed using multivariate regression analysis, in order to identify the patient-related, medication-related and environment-related factors that can increase the risk of hospitalisation, emergency department visits, readmission (notably due to MRPs), institutionalisation or early death. Furthermore, the profiles of polymedicated, home-dwelling OAMCC hospitalised or visiting the emergency department due to MRPs, and identified via multicluster analysis, will serve to guide the qualitative study and lead to a purposive sampling of polymedicated, home-dwelling OAMCC presenting with more risk factors. A draft of the cluster analysis strategy is available as an online supplementary file.

Phase 2: prospective patient-centred qualitative analysis

To meet the second and third objectives, a qualitative investigation, based on purposive sampling, will draw on work done in a feasibility study.[29] This qualitative investigation will consist of collecting and understanding the medication practices and experiences of OAMCC presenting with the risk factors identified in the first phase. The focus will be on identified OAMCC who were recently hospitalised and are at risk of hospital readmission. The older adult will be interviewed at home on two separate occasions. This methodology is a way to analyse changes in their medication practises and their experiences following their recent hospitalisation. The data

For peer review only - http://bmjopen.bmj.com/site/about/guidelines.xhtml
BMJ Open: first published as 10.1136/bmjopen-2019-030030 on 28 October 2019. Downloaded from http://bmjopen.bmj.com/ on November 12, 2019 at HES-SO Valais-Wallis - Fil?re Soins Infirmiers. Protected by copyright.
For peer review only

collection tools include a walking-interview[30] based on a medication journal and household photographs of where medication is stored. This allows us to focus on the tangible practices of OAMCC and contextualises them within the private space of their daily lives.

To discriminate the older adults' health profile, we will use the Resident Assessment Instrument-Home Care (RAI-HC) introduced by the Swiss Association for Home Care Services for all home care services in 2004. Based on a comprehensive geriatric assessment, the RAI-HC both allows for the establishment of an individualised care plan and generates quality indicators, plans resource use, optimises the medication management process by monitoring and documenting the number and types of medication and the persons involved in preparing medication, and regularly assesses adherence to the medication prescribed.[31] This instrument will provide information on the patient-related, medication-related and environment-related factors which may influence the occurrence of MRPs, and it will be used to recruit OAMCC at risk of or already presenting with MRPs.

Furthermore, we will also collect and analyse the perspectives of the formal and informal caregivers involved in medication management at home to cross-reference perspectives about this important dimension of care at home.

Research population

The profiles of the polymedicated OAMCC hospitalised/rehospitalised or consulting the emergency department, as identified in the retrospective investigation, will be used to select participants for the qualitative investigation. A theoretical, purposive sampling will be carried out. Based on Guest *et al*, the principal investigator will recruit about 30 polymedicated OAMCC (until saturation of data), all recently hospitalised (within the last 90 days) and at risk of hospital readmission.[32] For each OAMCC participant, an informal caregiver will also be integrated into the investigation. We defined informal caregivers as any family member, neighbour or friend assisting a dependent older adult with certain activities in their daily life. That assistance, help, care or physical presence must be given on a regular basis, for at least two basic activities or instrumental activities of daily living or to ensure patient safety, and for 6 months or more.[33] The informal caregiver will be included in the study if the recruited older adult identifies that person as being significant in their medication management and if they give informed written consent to participate.

Furthermore, a formal caregiver will be integrated into the investigation for each participant. Professional caregivers are those employed to provide professional healthcare services (ie, nurses, nursing assistants, general practitioners, pharmacists and social workers). They will be included in the study if the recruited OAMCC identifies them as the professional most involved in their medication management.

Table 1 presents the specific inclusion/exclusion criteria for each group of participants.

Participant recruitment

Polymedicated, home-dwelling OAMCC will be recruited via two paths so that all of the participants meet the eligibility criteria and fit corresponding profiles established in the quantitative phase. Some OAMCC will be receivers of care from Community Healthcare Centres and others will be functioning without that day-to-day support.

Table 1 Phase 2 inclusion and exclusion criteria

Participants	Inclusion criteria	Exclusion criteria
OAMCC	► Aged 65 or above<br>► Man or woman<br>► Hospitalised within the last 90 days<br>► Managing at least five different medications (prescribed and over-the-counter medications explored during recruitment)<br>► Suffering from multiple chronic conditions[4]<br>► Living alone or in a couple, in a rural or urban area<br>► With or without support from a Community Healthcare Centre	► Not able to speak and understand French
Informal caregiver	► Designated by the OAMCC as the most significant informal caregiver involved in medication management<br>► Aged 18 or above	► Not able to speak and understand French
Professional caregiver	► Designated by the OAMCC as having a key role in medication management	► Student<br>► Apprentice

OAMCC, older adults living with multiple chronic conditions.

For peer review only - http://bmjopen.bmj.com/site/about/guidelines.xhtml

BMJ Open: first published as 10.1136/bmjopen-2019-030030 on 28 October 2019. Downloaded from http://bmjopen.bmj.com/ on November 12, 2019 at HES-SO Valais-Wallis - Fil?re Soins Infirmiers. Protected by copyright.

- For OAMCC who do not receive support from a Community Healthcare Centre, recruitment will be based on variables in their patient files and carried out in collaboration with different nursing departments from the partner hospital.
- For OAMCC who do receive support from a Community Healthcare Centre, recruitment will be based on the clinical and health data documented in the RAI-HC and carried out in collaboration with community healthcare nurses from Sion Community Healthcare Centre.

Research nurses partnering the project, from a hospital or a Community Healthcare Centre, will briefly explain the study to the patient. Potential participants will be asked for permission to give their name to the researchers. The principal investigator will contact the older adult by telephone during the week following hospital discharge and ask for their agreement to participate in the study. In case of agreement, a first meeting will be organised at the older adult's home in the next few days. Participant recruitment will start in October 2019.

Data collection from OAMCC

During the first home meeting with the OAMCC, the principal investigator will provide all the study details and will suggest two semistructured interviews, each lasting about an hour, starting on the first meeting and spaced 2–3 weeks apart. According to participants' levels of tiredness, it may be necessary to subdivide the interviews. The older adult will be invited to sign the informed written consent form, allowing the researcher to collect sociodemographic and health data (RAI-HC and the patient's hospital records). Eligible home-dwelling OAMCC from both recruitment paths will be screened using the RAI-HC Minimal Data Set (MDS), which includes information on polymedication (section P), multiple chronic conditions (sections J and K) and recent hospitalisation (section Ac). Research team members trained on the RAI-HC will also carry out this evaluation for participants who do not have an RAI-HC. The following multidimensional clinical data will be retrieved from the RAI-HC MDS: cognitive status, hearing, vision, mood status, functional and physical status, continence, healthcare problems and nutritional state. The MDS will aid interviews with OAMCC and the exploration of the facilitators and barriers to daily medication management.

The first semistructured interview will collect the perspectives of OAMCC with regard to their medication management, the return home, information received about their treatment and its possible modifications, whether their opinions and preferences were taken into account in the prescription of medications, and the informal and professional caregivers involved. OAMCCs will be interviewed alone or with an informal caregiver, if necessary. The principal investigator will then ask the participant to complete a week-long medication journal,[34 35] either alone or with the help from informal or professional caregivers, emphasising that any information on daily medication routines is helpful, even if the OAMCC feels unable to complete the journal for the full 7 days. The instructions will mention the importance of noting all the medicines taken—those prescribed by general practitioners or specialist physicians, but also any others taken at their own initiative (over-the-counter medications). Participants will be asked to note their perceptions of and satisfaction with their treatment in a week-long medication journal. This will provide information on the daily routines associated with the participant's medication and will form the basis of the second interview.

The second interview will be based on the participant's medication journal and will take the form of a walking-interview[36] using household photographs.[35] The principal investigator will ask the participant to explain their medication practises while pointing out the locations within their home where drugs are stored, prepared and taken. The hypothesis underlying this methodology is that the physical presence of drugs promotes discussion.[37 38] We will identify and photograph, with the participants' agreement, the places where medication, contact details for medical professionals and other information are stored as well as the locations of any other objects involved in daily care practises. The collection and analysis of photographs provide a better understanding of the complexity of medication management in home settings. They help to capture the interviewee's concerns or strategies when they are pointed out to the interviewer. The interview guide will also investigate the issue of self-medication in order to reveal the extent and influence of this practice.

Data collection from informal caregivers

Sociodemographic data and information related to medication management will be collected. When possible and appropriate, a joint third interview[39] with the OAMCC and their principal informal caregiver[34] will be organised at the older adult's home 1–2 weeks after the walking interview. This type of interview provides access to the interactions between OAMCC and their informal caregivers with regard to medication management. We hypothesise that the main informal caregiver is deeply involved in the older adult's experience of medication management, but the caregiver's ideas about this may be similar to, overlapping with or different from those of an OAMCC.

Data collection from professional caregivers

A semistructured interview of about 1 hour will be conducted with a professional caregiver in order to explore their point of view on the OAMCC's medication management and other issues associated with the return home after hospitalisation. In agreement with the project's field partners and stakeholders, these interviews will take place in professionals' workplaces (Community Healthcare Centre, medical practice office or pharmacy), during working hours, 1–2 weeks after the interview with the OAMCC and their informal caregiver.

For peer review only

BMJ Open: first published as 10.1136/bmjopen-2019-030030 on 28 October 2019. Downloaded from http://bmjopen.bmj.com/ on November 12, 2019 at HES-SO Valais-Wallis - Fil?re Soins Infirmiers. Protected by copyright.

For peer review only - http://bmjopen.bmj.com/site/about/guidelines.xhtml

Qualitative data analyses

A database will be prepared using the RedCap software platform to record and store the participants' sociodemographic, health and interview data. Information on their health statuses will be collected using the RAI-HC data and will be analysed using the IBM-Statistical Package for Social Sciences V.25.0.

Data collected via the interviews will be examined according to an analytical plan that integrates and compares two different methods. First, thematic content analysis,[40 41] using NVivo V.12 software, will be used to identify the themes emerging from the data, and this will provide a rich, detailed account of the data set. Themes will be compared by different members of the analysis team until a consensus is reached. Second, lexicometric analysis, using Iramuteq software—a technique derived from the Alceste method[42]—will allow a very fine exploration, both within each interview and across the whole corpus of interviews, of the structures underlying the discourse. Each older adult's medication journal will be analysed and categorised according to the same principles as the interviews. The data collected from these documents will be put into perspective by the analysis of the interviews. In the final data analysis, links will be made between the interviews, the medication journal, the older adult's RAI-HC data and the photos of the medicines' locations.

Phase 3: development of a medication management model

Connecting retrospective and prospective findings, using an explanatory sequential design and participants' different perspectives, will contribute to a deep understanding of the current state of medication management practices of polymedicated, home-dwelling OAMCC. This mixed-methods study corresponds to the 'diagnostic' phase of the process of developing a Model of Care, as presented by the Agency for Clinical Innovation (ACI).[43] It will guide the 'solution design' phase—the next step in the creation of an innovative, integrated model for supporting medication management and preventing adverse health outcomes. In addition to the ACI's framework, the development of a proposed Medication Management Model will consider the quadruple aim of enhancing the patient's experience, improving population health, reducing costs and improving the working lives of healthcare providers.[44]

Finally, our mixed-methods research findings will be completed with those of an ongoing systematic review of Medication Management Models.[45]

The study phase outcomes are summarised in table 2.

Table 2 Outcomes for each study phase

Phase 1 outcomes	Patient-related, medication-related and environment-related factors which can increase the risk of hospitalisation, emergency department visits, hospital readmission (notably due to MRPs), institutionalisation or early death.<br>Profiles of polymedicated, home-dwelling OAMCC hospitalised or visiting the emergency department due to MRPs based on the previously identified patient-related, medication-related and environment-related factors.
Phase 2 outcomes	For OAMCC participants:<br>▶ Patient-related, medication-related and environment-related factors for MRPs (defined by phase 1's outcomes) extracted from the RAI-HC MDS and the patient's electronic hospital records (number and types of medication, multiple chronic conditions, recent hospitalisations, cognitive status, hearing, vision, mood status, functional and physical status, continence, healthcare problems and nutritional state).<br>▶ Medication practices and experiences of OAMCC following their recent hospitalisation, facilitators/barriers to medication management, informal and professional caregivers involved.<br>For informal caregivers:<br>▶ Sociodemographic profiles.<br>▶ Practices and experiences related to medication management.<br>For professional caregivers:<br>▶ Sociodemographic and professional profiles.<br>▶ Role and perspectives on OAMCC medication management.<br>▶ Coordination activities related to returning home after hospitalisation.
Phase 3 outcomes	Three first steps in the process of developing a Model of Care[43]:<br>▶ 'Project initiation'.<br>▶ 'Diagnostic'.<br>▶ 'Solution design' considering the quadruple aim.<br>Proposals for the Medication Management Model's 'Implementation' and 'Sustainability' steps[43] to support medication management and to prevent adverse health outcomes related to MRPs.

MDS, Minimal Data Set; MRPs, medication-related problems; OAMCC, older adults living with multiple chronic conditions; RAI-HC, Resident Assessment Instrument-Home Care.

Patient and public involvement

This study and the feasibility study on which it is based were developed in collaboration with representatives from a Community Healthcare Centre, a regional hospital, medical and pharmacy associations and an informal caregivers association. They shared their expertise on the study's relevance and the feasibility of data collection with the research team. Patients' priorities, experiences and preferences, collected during the feasibility study, were the drivers for the development of the research question and outcome measures.

A steering committee will involve these different actors at various stages in the project, both to contribute to data collection and to provide their expertise to the coconstruction of a Medication Management Model and its future implementation. As regards data collection, the hospital's medical informatics department will provide the appropriate data based on a data extraction protocol (phase 1) and the Community Healthcare

For peer review only - http://bmjopen.bmj.com/site/about/guidelines.xhtml

BMJ Open: first published as 10.1136/bmjopen-2019-030030 on 28 October 2019. Downloaded from http://bmjopen.bmj.com/ on November 12, 2019 at HES-SO Valais-Wallis - Fil?re Soins Infirmiers. Protected by copyright.

Centre will help with OAMCC recruitment and access to participants' RAI-HC and professional caregivers (phase 2).

Results will be disseminated to study participants through presentations to associations of patients and informal caregivers and at professional training sessions.

ETHICS AND DISSEMINATION

With the approval, the medical informatics department of partner hospital will provide the appropriate data for the retrospective phase based on a data extraction protocol. Extracted data will be delivered and stored in the ReDCap data platform via a secure coded data file. In coherence with the Data Management Plan submitted to the Swiss National Science Foundation, the collected data will be securely stored for future research.

The autonomy of the participants will be respected. Participation in the prospective phase in this research is free. It will be possible for participants to refuse to record the interview or to request the deletion of the recorded data. Participating in a structured effort to understand medication practises and the posthospital return home experience can contribute to improvements in health management in the community at large, and particularly in the area of home support.

Findings will be disseminated in peer-reviewed journals, professional conferences and other knowledge transfer activities with primary healthcare providers, hospital care units, informal caregiver and patient associations.

Author affiliations
[1]School of Health Sciences, HES-SO Valais-Wallis, Sion, Switzerland
[2]Institute of Biomedical Sciences Abel Salazar, University of Porto, Porto, Portugal
[3]Research Center for Psychology of Health, Aging and Sport Examination, University of Lausanne, Lausanne, Switzerland
[4]Service of Old Age Psychiatry, Lausanne University Hospital, Lausanne, Switzerland
[5]Swiss Centre of Expertise in the Social Sciences, University of Lausanne, FORS, Lausanne, Switzerland
[6]Higher School of Nursing of Porto, Porto, Portugal

Twitter Maria Manuela Martins @mmmartins1956

Contributors FP, PR and HV had the original idea. MSD, AVG, BW, MMM and HV provided conceptual and methodological expertise to the design of the research protocol. FP, PR and HV were major contributors to writing the manuscript. All authors read, edited and approved the final manuscript.

Funding This work is supported by the Swiss National Science Foundation grant number 407440_183434/1.

Competing interests None declared.

Patient consent for publication Not required.

Ethics approval Ethical approval has been obtained from the Human Research Ethics Committee of the Canton Vaud (2018-02196).

Provenance and peer review Not commissioned; externally peer reviewed.

Data availability statement Data are available upon reasonable request.

Open access This is an open access article distributed in accordance with the Creative Commons Attribution Non Commercial (CC BY-NC 4.0) license, which permits others to distribute, remix, adapt, build upon this work non-commercially, and license their derivative works on different terms, provided the original work is properly cited, appropriate credit is given, any changes made indicated, and the use is non-commercial. See: http://creativecommons.org/licenses/by-nc/4.0/.

ORCID iD
Filipa Pereira http://orcid.org/0000-0001-9207-4856

REFERENCES

1 Bachmann N, Burla L, Kohler D, *et al*. La santé en Suisse-Le point sur les maladies chroniques: Rapport national sur la santé 2015. Retrieved from Berne: & Older people's experiences of medicine changes on leaving hospital. Research in Social and Administrative Pharmacy 2015;10:791-800.
2 Marengoni A, Angleman S, Melis R, *et al*. Aging with multimorbidity: a systematic review of the literature. *Ageing Res Rev* 2011;10:430–9.
3 Valderas JM, Starfield B, Sibbald B, *et al*. Defining comorbidity: implications for understanding health and health services. *Ann Fam Med* 2009;7:357–63.
4 WHO. *Innovative care for chronic conditions: building blocks for action: global report*. Geneva: World Health Organization, 2002.
5 Maher RL, Hanlon J, Hajjar ER. Clinical consequences of polypharmacy in elderly. *Expert Opin Drug Saf* 2014;13:57–65.
6 Masnoon N, Shakib S, Kalisch-Ellett L, *et al*. What is polypharmacy? A systematic review of definitions. *BMC Geriatr* 2017;17:1–10.
7 Monégat M, Rococo E. *Polypharmacy: definitions, measurement and stakes involved. Review of the literature and measurement tests*, 2014.
8 Al Hamid A, Ghaleb M, Aljadhey H, *et al*. A systematic review of hospitalization resulting from medicine-related problems in adult patients. *Br J Clin Pharmacol* 2014;78:202–17.
9 Renom-Guiteras A, Meyer G, Thürmann PA. The EU(7)-PIM list: a list of potentially inappropriate medications for older people consented by experts from seven European countries. *Eur J Clin Pharmacol* 2015;71:861–75.
10 Nickel CH, Ruedinger JM, Messmer AS, *et al*. Drug - related emergency department visits by elderly patients presenting with non-specific complaints. *Scand J Trauma Resusc Emerg Med* 2013;21:15.
11 Fallis BA, Dhalla IA, Klemensberg J, *et al*. Primary medication non-adherence after discharge from a general internal medicine service. *PLoS One* 2013;8:e61735.
12 Bonnet-Zamponi D, d'Arailh L, Konrat C, *et al*. Drug-Related readmissions to medical units of older adults discharged from acute geriatric units: results of the optimization of medication in aged multicenter randomized controlled trial. *J Am Geriatr Soc* 2013;61:113–21.
13 Gilbert A, Roughead L, McDermott R, *et al*. Multiple Chronic Health Conditions in Older People: Implications for Health Policy Planning, Practitioners and Patients. University of South Australia 2013 [Ageing well ageing productively: people living with multiple chronic health conditions [1-48]. Available: https://www.unisa.edu.au/siteassets/episerver-6-files/global/health/sansom/documents/qumprc/multiple-chronic-health-conditions.pdf
14 Roughead EE, Vitry AI, Caughey GE, *et al*. Multimorbidity, care complexity and prescribing for the elderly. *Aging Health* 2011;7:695–705.
15 O'Quin KE, Semalulu T, Orom H. Elder and caregiver solutions to improve medication adherence. *Health Educ Res* 2015;30:323–35.
16 Gillespie R, Mullan J, Harrison L. Managing medications: the role of informal caregivers of older adults and people living with dementia. A review of the literature. *J Clin Nurs* 2014;23:3296–308.
17 Look KA, Stone JA. Medication management activities performed by informal caregivers of older adults. *Res Social Adm Pharm* 2018;14:418–26.
18 Reinhard SC, Levine C, Samis S. Home alone: family caregivers providing complex chronic care. *BMJ* 2012;41.
19 Pellegrin KL, Lee E, Uyeno R, *et al*. Potentially preventable medication-related hospitalizations: a clinical pharmacist approach to assessment, categorization, and quality improvement. *J Am Pharm Assoc* 2017;57:711–6.
20 Almanasreh E, Moles R, Chen TF. The medication reconciliation process and classification of discrepancies: a systematic review. *Br J Clin Pharmacol* 2016;82:645–58.
21 IHI. *How-to guide: prevent adverse drug events by implementing medication reconciliation*. Cambridge, Massachusetts, USA: Institute for Healthcare Improvement, 2011.
22 Barnsteiner JH, Reconciliation M. Medication reconciliation. In: Hughes RG, ed. *Patient safety and quality: an evidence-based Handbook for nurses*. Rockville (MD), 2008.

For peer review only

BMJ Open: first published as 10.1136/bmjopen-2019-030030 on 28 October 2019. Downloaded from http://bmjopen.bmj.com/ on November 12, 2019 at HES-SO Valais-Wallis - Fili?re Soins Infirmiers. Protected by copyright.

For peer review only - http://bmjopen.bmj.com/site/about/guidelines.xhtml

23 Creswell JW, Plano Clark VL. *Improving medication management in home care: issues and solutions*, 2011.
24 OECD. Health Status : Life expectancy, 2018. Available: https://stats.oecd.org/index.aspx?queryid=30114 [Accessed 1 Aug 2019].
25 Vassar M, Holzmann M. The retrospective chart review: important methodological considerations. *J Educ Eval Health Prof* 2013;10:12.
26 Motheral B, Brooks J, Clark MA, *et al*. A checklist for retrospective database studies--report of the ISPOR Task Force on Retrospective Databases. *Value Health* 2003;6:90–7.
27 Holzer B. SwissDRG – L'essentiel en bref. *Bulletin des médecins suisses* 2012;93:1079–81.
28 OFS. *Classification Suisse des interventions Chirurgicales (CHOP)*. Office fédéral de la statistique, 2018.
29 Roux P, Pereira F, Santiago-Delefosse M, *et al*. Medication practices and experiences of older adults discharged home from Hospital: a feasibility study protocol. *Patient Prefer Adherence* 2018;12:1055–63.
30 Evans J, Jones P. The walking interview: methodology, mobility and place. *Applied Geography* 2011;31:849–58.
31 Monod S, Büla C, Hongler T, *et al*. Le Resident Assessment Instrument-Home-Care (RAI-Domicile) : ce que le médecin de premier recours doit savoir. *Revue Médicale Suisse* 2011;7:2176–83.
32 Guest G, Bunce A, Johnson L. How many interviews are enough? *Field methods* 2006;18:59–82.
33 ÉD V. Commission consultative du soutien aux proches aidants. *État de Vaud* 2018.
34 Knight DA, Thompson D, Mathie E, *et al*. 'Seamless care? Just a list would have helped!' older people and their carer's experiences of support with medication on discharge home from hospital. *Health Expect* 2013;16:277–91.
35 Dew K, Chamberlain K, Hodgetts D, *et al*. Home as a hybrid centre of medication practice. *Sociol Health Illn* 2014;36:28–43.
36 Carpiano RM. Come take a walk with me: the "go-along" interview as a novel method for studying the implications of place for health and well-being. *Health Place* 2009;15:263–72.
37 Fainzang S. Les médicaments dans l'espace privé. *Anthropologie et Sociétés* 2003;27.
38 Haxaire C. « Calmer les nerfs » : automédication, observance et dépendance à l'égard des médicaments psychotropes. *Sciences sociales et santé* 2002;20:63–88.
39 Polak L, Green J. Using joint interviews to add analytic value. *Qual Health Res* 2016;26:1638–48.
40 Braun V, Clarke V. Using thematic analysis in psychology. *Qual Res Psychol* 2006;3:77–101.
41 Elo S, Kyngäs H. The qualitative content analysis process. *J Adv Nurs* 2008;62:107–15.
42 Ratinaud P, Déjean S. IRaMuTeQ: implémentation de la méthode ALCESTE d'analyse de texte dans un logiciel libre. *Modélisation Appliquée aux Sciences Humaines et Sociales* 2009:8–9.
43 ACI. *Understanding the process to develop a model of care: an ACI framework*. Chatswood: Agency for Clinical Innovation, 2013.
44 Bodenheimer T, Sinsky C. From triple to quadruple aim: care of the patient requires care of the provider. *Ann Fam Med* 2014;12:573–6.
45 Pereira F, Roux P, Rosselet Amoussou J, *et al*. Medication management models for Polymedicated home-dwelling older adults with multiple chronic conditions: protocol of a systematic review. *JMIR Res Protoc* 2019;8:e13582.

For peer review only

BMJ Open: first published as 10.1136/bmjopen-2019-030030 on 28 October 2019. Downloaded from http://bmjopen.bmj.com/ on November 12, 2019 at HES-SO Valais-Wallis - Fil?re Soins Infirmiers. Protected by copyright.

For peer review only - http://bmjopen.bmj.com/site/about/guidelines.xhtml

The RECORD statement – checklist of items, extended from the STROBE statement, that should be reported in observational studies using routinely collected health data.

	Item No.	STROBE items	Location in manuscript where items are reported	RECORD items	Location in manuscript where items are reported
**Title and abstract**					
	1	(a) Indicate the study's design with a commonly used term in the title or the abstract (b) Provide in the abstract an informative and balanced summary of what was done and what was found		RECORD 1.1: The type of data used should be specified in the title or abstract. When possible, the name of the databases used should be included.<br><br>RECORD 1.2: If applicable, the geographic region and timeframe within which the study took place should be reported in the title or abstract.<br><br>RECORD 1.3: If linkage between databases was conducted for the study, this should be clearly stated in the title or abstract.	Title<br>Abstract (lines 2-8)<br><br>Line 6<br><br>Lines 118-121<br><br>Not applicable, only one hospital register
**Introduction**					
Background rationale	2	Explain the scientific background and rationale for the investigation being reported			Lines 39-101
Objectives	3	State specific objectives, including any prespecified hypotheses			Lines 101-107
**Methods**					
Study Design	4	Present key elements of study design early in the paper			Lines 110-114
Setting	5	Describe the setting, locations, and relevant dates, including periods of recruitment, exposure, follow-up, and data collection			Lines 117-133

For peer review only

Participants	6	*(a) Cohort study* - Give the eligibility criteria, and the sources and methods of selection of participants. Describe methods of follow-up<br>*Case-control study* - Give the eligibility criteria, and the sources and methods of case ascertainment and control selection. Give the rationale for the choice of cases and controls<br>*Cross-sectional study* - Give the eligibility criteria, and the sources and methods of selection of participants<br><br>*(b) Cohort study* - For matched studies, give matching criteria and number of exposed and unexposed<br>*Case-control study* - For matched studies, give matching criteria and the number of controls per case		RECORD 6.1: The methods of study population selection (such as codes or algorithms used to identify subjects) should be listed in detail. If this is not possible, an explanation should be provided.<br><br>RECORD 6.2: Any validation studies of the codes or algorithms used to select the population should be referenced. If validation was conducted for this study and not published elsewhere, detailed methods and results should be provided.<br><br>RECORD 6.3: If the study involved linkage of databases, consider use of a flow diagram or other graphical display to demonstrate the data linkage process, including the number of individuals with linked data at each stage.	in press/ forthcoming JMIR article: https://www.ncbi.nlm.nih.gov/pubmed/management/validator/909A44E74F70/citations/?start=0<br><br>Not applicable, only one hospital register
Variables	7	Clearly define all outcomes, exposures, predictors, potential confounders, and effect modifiers. Give diagnostic criteria, if applicable.		RECORD 7.1: A complete list of codes and algorithms used to classify exposures, outcomes, confounders, and effect modifiers should be provided. If these cannot be reported, an explanation should be provided.	Lines 152-177 and in press/ forthcoming JMIR article: https://www.ncbi.nlm.nih.gov/pubmed/management/validator/909A44E74F70/citations/?start=0
Data sources/ measurement	8	For each variable of interest, give sources of data and details			Lines 122-128 and

For peer review only

		of methods of assessment (measurement). Describe comparability of assessment methods if there is more than one group			in press/ forthcoming JMIR article: https://www.ncbi.nlm.nih.gov/pubmed/management/validator/909A44E74F70/citations/?start=0
Bias	9	Describe any efforts to address potential sources of bias			Lines 180-200 and in press/ forthcoming JMIR article: https://www.ncbi.nlm.nih.gov/pubmed/management/validator/909A44E74F70/citations/?start=0
Study size	10	Explain how the study size was arrived at			Lines 141-144
Quantitative variables	11	Explain how quantitative variables were handled in the analyses. If applicable, describe which groupings were chosen, and why			Lines 139-150 and in press/ forthcoming JMIR article: https://www.ncbi.nlm.nih.gov/pubmed/management/validator/909A44E74F70/citations/?start=0

For peer review only

Statistical methods	12	(a) Describe all statistical methods, including those used to control for confounding<br>(b) Describe any methods used to examine subgroups and interactions<br>(c) Explain how missing data were addressed<br>(d) *Cohort study* - If applicable, explain how loss to follow-up was addressed<br>*Case-control study* - If applicable, explain how matching of cases and controls was addressed<br>*Cross-sectional study* - If applicable, describe analytical methods taking account of sampling strategy<br>(e) Describe any sensitivity analyses			Lines 180-200
Data access and cleaning methods		..		RECORD 12.1: Authors should describe the extent to which the investigators had access to the database population used to create the study population.<br><br>RECORD 12.2: Authors should provide information on the data cleaning methods used in the study.	Lines 118-122 and in press/ forthcoming JMIR article: https://www.ncbi.nlm.nih.gov/pubmed/management/validator/909A44E74F70/citations/?start=0
Linkage		..		RECORD 12.3: State whether the study included person-level, institutional-level, or other data linkage across two or more databases. The methods of linkage and methods of	in press/ forthcoming JMIR article: https://www.ncbi.nlm.nih.gov/pubm

For peer review only

				linkage quality evaluation should be provided.	ed/management/validator/909A44E74F70/citations/?start=0
**Results**					
Participants	13	(a) Report the numbers of individuals at each stage of the study (*e.g.*, numbers potentially eligible, examined for eligibility, confirmed eligible, included in the study, completing follow-up, and analysed)<br>(b) Give reasons for non-participation at each stage.<br>(c) Consider use of a flow diagram		RECORD 13.1: Describe in detail the selection of the persons included in the study (*i.e.,* study population selection) including filtering based on data quality, data availability and linkage. The selection of included persons can be described in the text and/or by means of the study flow diagram.	Lines 142-143
Descriptive data	14	(a) Give characteristics of study participants (*e.g.*, demographic, clinical, social) and information on exposures and potential confounders<br>(b) Indicate the number of participants with missing data for each variable of interest<br>(c) *Cohort study* - summarise follow-up time (*e.g.*, average and total amount)			Lines 152-177
Outcome data	15	*Cohort study* - Report numbers of outcome events or summary measures over time<br>*Case-control study* - Report numbers in each exposure category, or summary measures of exposure<br>*Cross-sectional study* - Report numbers of outcome events or summary measures			Lines 142-144

For peer review only

Main results	16	(a) Give unadjusted estimates and, if applicable, confounder-adjusted estimates and their precision (e.g., 95% confidence interval). Make clear which confounders were adjusted for and why they were included<br>(b) Report category boundaries when continuous variables were categorized<br>(c) If relevant, consider translating estimates of relative risk into absolute risk for a meaningful time period			Lines 207-285
Other analyses	17	Report other analyses done—e.g., analyses of subgroups and interactions, and sensitivity analyses			Lines 207-285
**Discussion**					
Key results	18	Summarise key results with reference to study objectives			Lines 289-294
Limitations	19	Discuss limitations of the study, taking into account sources of potential bias or imprecision. Discuss both direction and magnitude of any potential bias		RECORD 19.1: Discuss the implications of using data that were not created or collected to answer the specific research question(s). Include discussion of misclassification bias, unmeasured confounding, missing data, and changing eligibility over time, as they pertain to the study being reported.	Lines 369-378
Interpretation	20	Give a cautious overall interpretation of results considering objectives, limitations, multiplicity of analyses, results from similar studies, and other relevant evidence			Lines 294-361

For peer review only

Generalisability	21	Discuss the generalisability (external validity) of the study results			Lines 349-355
**Other Information**					
Funding	22	Give the source of funding and the role of the funders for the present study and, if applicable, for the original study on which the present article is based			Line 399
Accessibility of protocol, raw data, and programming code		..		RECORD 22.1: Authors should provide information on how to access any supplemental information such as the study protocol, raw data, or programming code.	in press/ forthcoming JMIR article: https://www.ncbi.nlm.nih.gov/pubmed/management/validator/909A44E74F70/citations/?start=0

*Reference: Benchimol EI, Smeeth L, Guttmann A, Harron K, Moher D, Petersen I, Sørensen HT, von Elm E, Langan SM, the RECORD Working Committee. The REporting of studies Conducted using Observational Routinely-collected health Data (RECORD) Statement. *PLoS Medicine* 2015; in press.

*Checklist is protected under Creative Commons Attribution (CC BY) license.

For peer review only

BMJ Open

Risks of 30-day hospital readmission associated with medical conditions and drug regimens of polymedicated, older inpatients discharged home: a registry-based cohort study

Journal:	*BMJ Open*
Manuscript ID	bmjopen-2021-052755.R1
Article Type:	Original research
Date Submitted by the Author:	19-Jun-2021
Complete List of Authors:	Pereira, Filipa; University of Porto, Institute of Biomedical Sciences Abel Salazar; HES-SO University of Applied Sciences and Arts Western Switzerland, School of Health Sciences<br>Verloo, Henk; HES-SO University of Applied Sciences and Arts Western Switzerland, School of Health Sciences; Lausanne University Hospital, Service of Old Age Psychiatry<br>Zhivko, Taushanov; University of Geneva<br>Di Giovanni, Saviana; HES-SO University of Applied Sciences and Arts Western Switzerland, School of Health Sciences; Pharmacy Benu Tavil-Chatton<br>Meyer-Massetti, Carla; University of Bern, Institute for Primary Health Care<br>Von-Gunten, Armin; Lausanne University Hospital, Service of Old Age Psychiatry<br>Martins, Maria Manuela; Porto Higher School of Nursing; University of Porto, Institute of Biomedical Sciences Abel Salazar<br>Wernli, Boris; University of Lausanne, FORS, Swiss Centre of Expertise in the Social Sciences
**Primary Subject Heading**:	Geriatric medicine
Secondary Subject Heading:	Epidemiology, Health informatics
Keywords:	GERIATRIC MEDICINE, CLINICAL PHARMACOLOGY, EPIDEMIOLOGY

SCHOLARONE™
Manuscripts

I, the Submitting Author has the right to grant and does grant on behalf of all authors of the Work (as defined in the below author licence), an exclusive licence and/or a non-exclusive licence for contributions from authors who are: i) UK Crown employees; ii) where BMJ has agreed a CC-BY licence shall apply, and/or iii) in accordance with the terms applicable for US Federal Government officers or employees acting as part of their official duties; on a worldwide, perpetual, irrevocable, royalty-free basis to BMJ Publishing Group Ltd ("BMJ") its licensees and where the relevant Journal is co-owned by BMJ to the co-owners of the Journal, to publish the Work in this journal and any other BMJ products and to exploit all rights, as set out in our licence.

The Submitting Author accepts and understands that any supply made under these terms is made by BMJ to the Submitting Author unless you are acting as an employee on behalf of your employer or a postgraduate student of an affiliated institution which is paying any applicable article publishing charge ("APC") for Open Access articles. Where the Submitting Author wishes to make the Work available on an Open Access basis (and intends to pay the relevant APC), the terms of reuse of such Open Access shall be governed by a Creative Commons licence – details of these licences and which Creative Commons licence will apply to this Work are set out in our licence referred to above.

Other than as permitted in any relevant BMJ Author's Self Archiving Policies, I confirm this Work has not been accepted for publication elsewhere, is not being considered for publication elsewhere and does not duplicate material already published. I confirm all authors consent to publication of this Work and authorise the granting of this licence.

For peer review only

Risks of 30-day hospital readmission associated with medical conditions and drug regimens of polymedicated, older inpatients discharged home: a registry-based cohort study

---

Pereira Filipa[1], Verloo Henk[2], Taushanov Zhivko[3], Di Giovanni Saviana[4], Meyer-Massetti Carla[5], von Gunten Armin[6], Martins Maria Manuela[7], Wernli Boris[8]

[1] Institute of Biomedical Sciences Abel Salazar, University of Porto
Rua de Jorge Viterbo Ferreira, 228 4050-313 Porto, Portugal
School of Health Sciences, HES-SO University of Applied Sciences and Arts Western Switzerland
5, Chemin de l'Agasse, CH-1950 Sion, Switzerland
Email: filipa.pereira@hevs.ch; Phone: +41 78 666 17 00; Fax: +41 27 606 84 00
ORCID: https://orcid.org/0000-0001-9207-4856

[2] School of Health Sciences, HES-SO University of Applied Sciences and Arts Western Switzerland
5, Chemin de l'Agasse, CH-1950 Sion, Switzerland
Service of Old Age Psychiatry, Lausanne University Hospital
Email: henk.verloo@hevs.ch; Phone: +41 27 606 84 34
ORCID: http://orcid.org/0000-0002-5375-3255

[3] University of Geneva
CH-1205 Geneva, Switzerland
Email: zhivko.taushanov@unige.ch
ORCID: https://orcid.org/0000-0002-3798-757X

[4] School of Health Sciences, HES-SO University of Applied Sciences and Arts Western Switzerland
5, Chemin de l'Agasse, CH-1950 Sion, Switzerland
Pharmacy Benu Tavil-Chatton
Grand rue 11, CH-1110 Morges, Switzerland
Email: saviana.digiovanni@gmail.com

[5] Institute for Primary Health Care, University of Bern
Mittelstrasse 43, CH-3012 Bern, Switzerland
Email: carla.meyer-massetti@biham.unibe.ch
ORCID: https://orcid.org/0000-0002-3523-5729

[6] Service of Old Age Psychiatry,

For peer review only

Lausanne University Hospital
Route de Cery 60, 1008 Prilly, Switzerland
Email: armin.von-gunten@chuv.ch; Phone: +41 21 314 52 67
ORCID: https://orcid.org/0000-0001-7852-3803

[7] Higher School of Nursing of Porto
Institute of Biomedical Sciences Abel Salazar, University of Porto
Rua Dr. António Bernardino de Almeida
4200-072 Porto, Portugal
Email: mmartins@esenf.pt; Phone: +351 22 507 35 00
ORCID: https://orcid.org/0000-0003-1527-9940

[8] FORS, Swiss Centre of Expertise in the Social Sciences, University of Lausanne
Géopolis, CH-1015 Lausanne, Switzerland
Email: boris.wernli@fors.unil.ch; Phone: +41 21 692 37 23
ORCID: https://orcid.org/0000-0002-5567-1317

Corresponding author:
Pereira F
Institute of Biomedical Sciences Abel Salazar, University of Porto
Rua de Jorge Viterbo Ferreira, 228 4050-313 Porto, Portugal
School of Health Sciences, HES-SO University of Applied Sciences and Arts Western Switzerland
5, Chemin de l'Agasse, CH-1950 Sion, Switzerland
Email: filipa.pereira@hevs.ch; Phone: +41 78 666 17 00; Fax: +41 27 606 84 00
ORCID: https://orcid.org/0000-0001-9207-4856

For peer review only

Abstract

Objectives: The present study analysed four years of a hospital register (2015–2018) to determine the risks of 30-day hospital readmission associated with the medical conditions and drug regimens of polymedicated, older inpatients discharged home.

Design: Registry-based cohort study.

Setting: Valais Hospital—a public general hospital centre in the French-speaking part of Switzerland.

Participants: We explored the electronic records of 20,422 inpatient stays by polymedicated, home-dwelling older adults held in the hospital's patient register. We identified 13,802 hospital readmissions involving 8,878 separate patients over 64 years old.

Outcome measures: Sociodemographic characteristics, medical conditions and drug regimen data associated with the risk of readmission within 30 days of discharge.

Results: The overall 30-day hospital readmission rate was 7.8%. Adjusted multivariate analyses revealed increased risks of hospital readmission for patients with longer hospital lengths of stay (OR = 1.014 per additional day; 95% CI: 1.006–1.021), impaired mobility (OR = 1.218 ; 95% CI: 1.039–1.427), multimorbidity (OR = 1.419 per additional ICD-10 condition; 95% CI: 1.282–1.572), tumoural disease (OR = 2.538; 95% CI: 2.089–3.082), polypharmacy (OR = 1.043 per additional drug prescribed; 95% CI: 1.028–1.058), and certain specific drugs, including antiemetics and antinauseants (OR = 3.216 per additional drug unit taken; 95% CI: 1.842–5.617), antihypertensives (OR = 1.771; 95% CI: 1.287–2.438), drugs for functional gastrointestinal disorders (OR = 1.424; 95% CI: 1.166–1.739), systemic hormonal preparations (OR = 1.207; 95% CI: 1.052–1.385), and vitamins (OR = 1.201; 95% CI: 1.049–1.374), as well as the concurrent use of beta-blocking agents and drugs for acid-related disorders (OR = 1.367; 95% CI: 1.046–1.788). **Conclusions:** Thirty-day hospital readmission risks were associated with longer hospital length of stay, health disorders, polypharmacy and drug regimens. The drug regimen patterns increasing the risk of hospital readmission were very heterogeneous. Further research is needed to explore hospital readmissions caused solely by specific drugs and drug–drug interactions.

Keywords: polypharmacy; odds ratio; logistic regression; hospital register; ATC Classification System; adverse-drug events; hospital readmission.

Strengths and limitations of this study:

- The records of 20,422 hospitalisations involving 8,878 different polymedicated home-dwelling older patients readmitted to hospital at least once were studied to determine the risks of 30-day hospital readmission.
- The study included four years' data from a comprehensive hospital register (2015–2018).
- A whole series of sociodemographic and clinical parameters, medical conditions and prescribed drugs were used to predict the probability of hospital readmission.
- Analyses were correlational and causality was not explored.

For peer review only - http://bmjopen.bmj.com/site/about/guidelines.xhtml
For peer review only

- Although the study considered statistical associations between drugs and hospital readmissions, it did not consider clinically diagnosed drug–drug interactions.

Introduction

Longitudinal studies have demonstrated that approximately 20% of the home-dwelling older adults supported by home health-care services experienced hospital readmission within 30 days of their discharge (1-3). For many older adults, readmission to an acute hospital is associated with a functional decline that has not always recovered by the time they are discharged (4). However, the systematic review by Hansen *et al*. revealed wide-ranging estimates (5%–79%) of how many hospital readmissions were preventable (5). The period between hospital discharge and readmission has not always been clearly stated in the literature, ranging from 30 days to 3 years. However, 30 days is the most frequently used in public health policy when measuring health-care system performance (6-8).

Numerous determinants have been identified and associated with hospital readmissions, e.g. sociodemographic and individual characteristics, multimorbidity and medical events (9, 10). A substantial risk of 30-day hospital readmission has been associated with older inpatients treated for different diseases and surgical interventions involving hip fracture, cancer, bypass, acute cardiovascular events or complex surgery (11). The reasons for hospital readmission after a surgical intervention are often not directly related to the surgery itself but rather to underlying chronic health conditions (12). Thus, chronic diseases may play an important role in readmission risk, independently of the reason for the initial hospitalisation (13, 14). Older adults' chronic diseases are not isolated health conditions; they can influence each other, and treatment for one disease may adversely affect another (15). For all these reasons, patterns of 30-day hospital readmissions may be very complex (16).

Multimorbidity, in the case of two or more diseases (17, 18), may require taking multiple medicines (19), known as polypharmacy (PP) when daily intake involves five or more drugs (20). Increasing incidences of multimorbidity with age, and consequently PP, add to the complexity of managing older inpatients' drug prescriptions, particularly at hospital discharge (21, 22). PP and inadequate drug management are significant risk factors for adverse drug events (ADEs)—the most common post-discharge complications—alongside hospital-acquired infections and procedural complications (23, 24). ADEs resulting from inappropriate drug prescribing, discrepancies between prescribed and current regimens, poor adherence and the inadequate surveillance of adverse effects frequently lead to hospital admissions, readmissions (8) and other undesirable consequences such as increased morbidity, decreased autonomy, institutionalisation and even early death (25, 26). A systematic review by Morabet *et al.* indicated ADE rates of 18%–38% after hospital discharge and 4.5%–24% hospital readmission rates due to those ADEs (27). Because older adults use more drugs, they are at a greater risk of drug-related readmission. Numerous studies have found that nearly 30% of older inpatients experienced ADEs within three weeks of hospital discharge, almost three-quarters of which could have been prevented or lessened (10, 28, 29).

For peer review only - http://bmjopen.bmj.com/site/about/guidelines.xhtml

Despite the significant overall impact of ADEs on hospital readmission rates, little is known about hospital readmission risk's associations with medical conditions and drug regimens (30, 31). Morabet *et al*. revealed the high prevalence of antibiotics, diuretics, vitamin K antagonists, opioids, antidiabetics, anti-cancer drugs, antihypertensives, digitalis glycosides, corticosteroids and psychotropic drugs in drug-related hospital readmissions (27). Samoy *et al*. reported that anticoagulants, hypoglycaemics, beta-blocking agents, antidepressants, calcium channel blockers and lenograstim were associated with high risks of hospital readmission (32). A retrospective patient record study by Teymoorian *et al*. reported that anticoagulants and antiplatelet agents, diuretics and antihypertensives, and opioids were associated with a high risk of persons aged 80 years old or more being readmitted to hospital within 30 days (33). Blanc *et al*. reported the readmission scores of different drugs in a large sample of 10,374 adult hospital admissions in general medicine. Taking beta-blocking agents, calcium channel blockers, diuretics, hypoglycaemic drugs or opioids was a significant risk for 30-day readmission (9).

Besides higher risks of drug-related hospital readmission, some studies have also investigated associations between combining drugs—a common practice when treating complex diseases or co-existing medical conditions—and drug-related hospital readmissions. Although using multiple drugs may be good clinical practice and compliant with guidelines for treating certain diseases, one significant consequence of combining drugs is that patients face much higher risks of ADEs, which can be caused by drug–drug interactions (34-36). ADEs can emerge because a drug's pharmacokinetics and pharmacodynamics change if taken with another drug (36). Moura *et al*. found that participants with potential drug–drug interactions on their prescription list had a 2.4 times higher adjusted odds ratio (OR) of being readmitted (37).

Even though some studies have reported high numbers of readmissions among home-dwelling older patients for a variety of drugs (38), this health issue was mostly investigated using prospective or cross-sectional studies with small samples. More insight is needed into patterns of drug-related hospital readmissions and risk factors in order to design better interventions for addressing ADEs (39, 40). As part of a broader project (41), the present study's goal was to use hospital register data to prioritise risk factors for hospital readmission. We hypothesised that sociodemographic characteristics, medical conditions (defined using the WHO's International Classification of Diseases, tenth revision: ICD-10, and the Swiss Classification of Surgical Interventions: CHOP), and drug prescriptions (based on the WHO's Anatomical Therapeutic Chemical (ATC) Classification System) were significant risk factors for 30-day hospital readmission for discharged older adults.

Material and Methods

Study Design

This longitudinal study (2015–2018) used data on a population cohort taken from a hospital registry composed of 140 variables. These were used to investigate the associations between the risks of 30-day hospital readmission and the medical conditions and drug regimens of polymedicated older inpatients

For peer review only - http://bmjopen.bmj.com/site/about/guidelines.xhtml

discharged home. The study was performed with close regard to the REporting of studies Conducted using Observational Routinely collected health Data (RECORD) statement (42).

Population and Data Collection

Our custom, four-year, registry-based dataset included polymedicated inpatients (five or more drugs prescribed at hospital discharge), aged 65 years old or more, living in their own homes and hospitalised at least once at the Valais Hospital (a public general hospital in the French-speaking part of Switzerland). This specific population was selected because of its increased risk of hospital readmission (10, 28, 29). Older inpatients hospitalised once only or who died during hospitalisation were excluded, as were those hospitalised for fewer than 24 hours (the criterion to count as "hospitalised" in Switzerland). Valais Hospital's register contains a comprehensive electronic health record composed of 140 variables routinely collected during hospital stays. However, no electronic patient records were available for adult psychiatry for 2015–2018. The extracted patient data contained sociodemographic characteristics, medical and surgical diagnoses, routinely assessed clinical data (such as gait, falls risk or hearing) from hospitalised patients with at least five prescribed drugs at discharge. Medical and surgical diagnoses were coded based on the ICD-10 and CHOP (43). Drug classification was based on the WHO's ATC Classification System (44).

The strategy for transforming and synthesising the data extracted from the register's multiple dataset sources was based on Olsen's register-based methodological considerations (45) and has been documented elsewhere (46). Our dataset was composed of 20,422 hospital admission records running from January 2015 to December 2018, with similar numbers of annual hospital admissions: 5134, 5095, 5125, and 5068, respectively.

Dataset Customisation for Predictive Analysis

The dataset was recoded and customised to identify the frequency of older patients' hospital admissions. Each subject's unique identifier was used to distinguish their different hospital stays from 2015–2018. The dataset included 13,802 readmissions involving 8,878 different older inpatients discharged home, readmitted to hospital within 30 days and whose data were complete (no missing values). Sociodemographic and clinical data were considered independent variables and used to compute the predictive models. Readmission following discharge home was defined as the dependent variable of interest and was dichotomised (0 = no, 1 = yes) based on 30-day readmission between 2015 and 2018. Furthermore, the custom dataset was composed of six clinical clusters based on agglomerative hierarchical clustering methods for identifying clinically relevant characteristics and representing older inpatients' health status. Medical status and drugs data were recoded and copied to an exploitable population database (46).

Sociodemographic Variables and Length of Stay

The sociodemographic data set—almost exclusively composed of ordinal variables—included two categorical variables (sex and place of discharge from hospital) and three continuous variables (age and

For peer review only - http://bmjopen.bmj.com/site/about/guidelines.xhtml

admission and discharge dates). Sex and age were included in the analysis as sociodemographic control variables. Age was considered a continuous variable as its progressive impact has been proven in preliminary investigations and previous studies (47).

Health Variables

Numerous variables were used to describe older patients' health status during each hospital stay. The health dataset was composed of 23 categorical variables: 21 measured as ordinal variables (mobility, changing position, falls in the last year, etc.) and two measured as nominal variables (altered gait and chronic pain). A cleaner, better-structured dataset—composed of hierarchical clusters—was obtained in a previous study combining empirical and best-practice statistical approaches (46). Three of six preliminarily computed hierarchical clusters were included in the modeling analysis as confounding variables: the mobility cluster, the dependency in the activities of daily living cluster and the mental state cluster (46). These three clusters were selected because of their significant contributions to hospital readmissions (48-50). The dataset of medical information was composed of patients' principal medical diagnosis and four secondary medical diagnoses, based on the ICD-10. Finally, the year of hospitalisation was introduced as a control variable, based on the fact that earlier admission to hospital during this period led to a higher probability of unplanned readmissions during the entire period covered.

Included Drugs

The hospital dataset showed that discharged patients had been prescribed 2,370 different drugs. Drug prescriptions were considered continuous, classified according to the WHO's ATC Classification System (51) and then included in the predictive model as independent variables. To ensure robust statistical results, the model only included drug categories prescribed to at least 30 inpatients who were readmitted within 30 days. Supplementary File 1 presents the prescribed ATC classified drugs included in the predictive model as independent variables.

For statistical purposes, drug–drug interactions between different ATC drug classes (51) were operationalised as dichotomised variables (0 = no simultaneous use of drugs from both classes, 1 = simultaneous use of drugs from both classes) and added to the previous model. Drug class interactions were selected based on a literature review, significant ORs and expert opinions (52).

Data analysis strategy

Data were extracted into a Microsoft Excel spreadsheet (Microsoft, Redmond, Washington, United States) and then imported into SPSS software, version 26.0 (IBM Corp, Armonk, New York, United States). We examined statistical associations between hospital readmissions and patient age and sex, LOS, principal and related ICD-10 diagnoses, CHOP interventions and drug prescriptions during hospitalisations. A causality analysis between those variables was impossible given our retrospective data collection method, our inability to calculate the time between drug intake and readmission, and the potential drug changes between hospitalisation sequences. We conducted a bivariate analysis relating the independent variables to 30-day readmission after discharge home from 2015–2018. Next, we calculated a series of multilevel logistic regression models for binary outcomes explaining the readmissions, within

For peer review only - http://bmjopen.bmj.com/site/about/guidelines.xhtml

30 days, of patients discharged home (0 = no, 1 = yes). These hierarchical models included two levels: the first level concerned hospital stays themselves, nested in the second level, that of individuals. Firstly, we computed a baseline multilevel binary logistic regression model to estimate how sets of predictors influenced the probability of 30-day hospital readmission, which included individuals' characteristics, health conditions and hospital LOS. Secondly, we completed this baseline model with the drugs prescribed to older inpatients on their discharge home. Finally, to that baseline model completed with prescribed drugs, we added the known drug–drug interactions between different ATC drug classes, based on a literature review and expert opinions. The model computed each predictor's impact, other things being equal, by estimating its net impact, controlling for other factors (adjusted ORs). The model also considered correlations between each subject's different variables, which were generally not independent (53). The model's random intercept design allowed each individual's intercept to vary, assuming that some unmeasured traits remained stable over time and allowing a better estimation of the model's parameters. The estimated parameters, on the other hand, had the same effect on every subject. Since the data were based on the whole population—not a sample—of polymedicated older inpatients discharged home from the Valais Hospital, the ORs' confidence intervals and statistical tests were used to indicate the robustness of relationships (they usually only make sense for statistical inference).

~~In a second stage,~~

Patients and public involvement

Patients were not involved in the development of the research questions, study design, outcome measures and conduct of the study.

Results

Descriptive results

The electronic records of 20,422 inpatient stays by polymedicated, home-dwelling older adults included the 13,802 hospital readmissions of 8,878 different older inpatients previously discharged home—an average of 1.55 inpatient hospital readmissions. The total sample's mean age was 77.77 years old (SD = 7.48), and 57% were men (Table 1). The average hospital LOS was 8.44 days (SD = 7.58). At discharge, 25% of the sample had impaired mobility, 4% were impaired in their activities of daily living and 4% showed mental impairment. Our sample population averaged 4.58 (SD = 0.92) ICD-10 diagnoses and 1.83 (SD = 1.76) surgical interventions (CHOP) performed during hospitalisation. The selected medical diagnoses distinguished patients affected by circulatory (24%), infectious (3%) and respiratory diseases (11%), as well as trauma (8%) and tumours (11%). On average, 8.95 (SD = 3.24) drugs were prescribed per patient at hospital discharge.

[Insert Table 1]

For peer review only - http://bmjopen.bmj.com/site/about/guidelines.xhtml

Associations between Thirty-day Hospital Readmission Risk and Sociodemographic Characteristics and Medical Conditions

The rate of 30-day hospital readmission for older patients discharged home was 7.8%. Bivariate associations with chi-square tests showed significant differences between older inpatients' sociodemographic characteristics and medical conditions (Table 2). Men showed a slightly higher proportion of 30-day hospital readmissions than women (8.2% vs 7.3%). However, age did not significantly affect the probability of 30-day readmission. More readmissions were also seen among older patients with a circulatory disease (8.2% vs 6.5%), those not affected by trauma (8.0% vs 5.8%), and especially those with a tumour (15.1% vs 6.9%). Multimorbidity also increased the risk of 30-day hospital readmissions—from 1.5% for older patients with a single ICD-10 condition to 8.8% for those with five.

[Insert Table 2]

Associations between Thirty-day Hospital Readmission Risk and Drugs

On average, older patients readmitted within 30 days had more prescribed drugs than those who were not readmitted (9.95 drugs vs 8.87). We found a linear relationship between the 30-day readmission rate and the average number of prescribed drugs ($p > 0.001$), which supported the absence of a cut-off point in this relationship (Figure 1).

[Insert Figure 1]

Among the most robust statistical associations (chi-square tests) with 30-day hospital readmissions involved the classes of drugs including antineoplasics and immunomodulators (12.6% vs 7.6% for those not treated with them) and taking antiemetics and antinauseants (27.7% vs 7.7%). There was also a higher risk of 30-day hospital readmission among older inpatients taking drugs for functional gastrointestinal disorders (13.4% vs 7.4%) and antihypertensives (14.1% vs 7.7%) (Table 3).

[Insert Table 3]

Baseline Multivariate Model

A baseline, multivariate logistic regression model including older patients' sociodemographic and clinical variables, but not their prescribed drugs at discharge, was computed to predict 30-day hospital readmission after discharge home (Table 4). Neither sex nor age had a significant impact. On the contrary, LOS had a significant impact (OR = 1.014 for each additional day; 95% CI: 1.006–1.021), as did mobility (OR = 1.218 for older patients with an impaired mobility status; 95% CI: 1.039–1.427). Dependence in the activities of daily living and mental health status showed no influence. Concerning diagnoses measured in the ICD-10, we found that older patients with a tumoural disease (OR = 2.538; 95% CI: 2.089–3.082) were much more susceptible to 30-day hospital readmission. Patients with circulatory pathologies showed no difference from the reference category (OR = 0.938; 95% CI: 0.783–1.124), and nor did those with respiratory problems (OR = 1.100; 95% CI: 0.875–1.382), trauma (OR = 0.847; 95% CI: 0.633–1.134) or infection-related problems (OR = 1.381; 95% CI: 0.964–1.977; $p = 0.078$). Multimorbidity predicted a higher probability of readmission (OR = 1.419 per additional ICD-

For peer review only - http://bmjopen.bmj.com/site/about/guidelines.xhtml

10 condition; 95% CI: 1.282–1.572), whereas the number of surgical procedures had no noticeable impact (OR = 0.978; 95% CI: 0.938–1.020). The year of hospital stay did have an impact, however, as the earlier the hospitalisation during the four years under review, the higher the probability of readmission (OR = 0.933 per additional year; 95% CI: 0.880–0.990).

Some variables that were non-significant in bivariate analyses became significant in multivariate analyses. This was because the results of multivariate analyses were controlled by all the other parameters and interpretations were made with "other things being equal". Also, the composition of subgroups could be very different in some bivariate analyses.

[Insert Table 4]

Prediction of 30-day Hospital Readmission and Drug Prescriptions

Table 5 presents the baseline logistic regression model completed with the drugs prescribed to older patients at discharge home that were significantly associated ($p = < 0.05$) with 30-day hospital readmission. It was not possible to introduce the total number of drugs prescribed jointly in this model because of their collinearity with other drug variables. Non-significant drugs and other variables have been omitted from Table 3 in order to simplify the presentation. The probabilities of 30-day hospital readmission are presented in descending order of discharged older patients' ORs for each additional unit of the drugs in question. Intake of antiemetics and antinauseants was very strongly linked to 30-day readmission (OR = 3.216 for each additional drug unit taken; 95% CI: 1.842–5.617), as were those of antihypertensives (OR = 1.771; 95%CI: 1.287–2.438), gastrointestinal drugs (OR = 1.424; 95% CI: 1.166–1.739), systemic hormonal preparations (OR = 1.207; 95% CI: 1.052–1.385) and vitamins (OR = 1.201; 95% CI: 1.049–1.374). On the contrary, the intake of lipid-modifying agents was associated with a decrease in 30-day hospital readmissions (OR = 0.841 for each drug from this class prescribed; 95% CI: 0.732–0.967).

[Insert Table 5]

Drug Interactions and 30-day Hospital Readmissions

The model considered drug class interactions for the: 1) cardiovascular system * central nervous system, gastrointestinal system, and metabolism * cardiovascular system; 2) gastrointestinal system and metabolism * central nervous system; 3) cardiovascular system * anti-infectives; and 4) central nervous system * anti-infectives. The analysis was carried out controlling for the basic model's variables (Table 4), and the table reports the ORs for each additional unit of the statistically significant drugs in question, as well as for significant drug interactions. Antiemetics and antinauseants were very strongly associated with 30-day readmission (OR = 3.222; 95% CI: 1.844–5.630), as were drugs regulating the gastrointestinal tract (OR = 1.428; 95% CI: 1.169–1.744) and systemic hormones (OR = 1.210; 95% CI: 1.054–1.390). The joint intake of beta-blocking agents and drugs for acid-related disorders was significantly associated with 30-day hospital readmission (OR = 1.367; 95% CI: 1.046–1.788); this is the only significant drug interaction in Table 4. On the contrary, lipid-modifying agents were associated with

For peer review only - http://bmjopen.bmj.com/site/about/guidelines.xhtml

lower 30-day hospital readmission (OR = 0.838), as were substances acting on the renin–angiotensin system (OR = 0.892; 95% CI: 0.796–0.999) (Table 6).

[Insert Table 6]

Discussion

The present study examined the records of 20,422 hospitalisations involving polymedicated home-dwelling older patients, eventually discharged home, for the risk of 30-day hospital readmission. These records were held in four years of a comprehensive hospital register. The 8,878 individual older patients readmitted to the Valais Hospital showed a 30-day hospital readmission rate of almost 8%, corroborating previously published all-cause hospital readmission rates among home-dwelling older patients (9, 27). However, Jencks *et al*. (2009) found a much higher 30-day readmission rate, reaching almost 20% among discharged older patients who had been hospitalised in acute medicine and surgery wards (3). As a bivariate association, multimorbid men were at a significantly higher risk of readmission than multimorbid women; however, in the adjusted multivariate analysis, that significance disappeared. Medical conditions, PP and multiple classes of prescribed drugs were all associated with higher 30-day readmission rates, in line with previous studies (27, 54-56).

Our study found no significant differences in the risks of 30-day hospital readmission for men and women. However, some previous research found that men were more likely to forget to take their drugs or to not apply the changed drug dosages prescribed by their family physician, consequently increasing their risk of hospital readmission for drug-related problems (57). Opposite results were found in a population-based study by Manteufel *et al*.(58), with women being less likely than men to properly adhere to their drug prescriptions. These differences may indicate a need for more personalised drug prescription and drug management to improve clinical outcomes. Further research should explore associations between different types of drugs and sex (58, 59), but this topic was beyond the present study's scope. Another interesting issue regarding sex differences in hospital readmission rates is the study window. Some studies found higher rates among men than among women below three-month readmissions. More extended time windows (e.g. one year) revealed no significant sex differences (54, 60). An analysis of our dataset using a more extended readmission window might clarify this point and provide complementary knowledge about sex-associated hospital readmissions.

Our results indicated that ageing was not a risk factor for increased 30-day hospital readmission, in line with some previous publications (55, 61). However, other research findings demonstrated that age was only positively associated with the likelihood of readmission up to 74 years old; above that, there no longer appeared to be any significant relationship between age and readmission (62, 63). These contrasting results may be explained by the studies' designs, country settings, the ages of their research populations or the medical conditions included (55, 62, 64).

Longer hospital stays were also associated with a higher risk of hospital readmission, in line with a cohort study by Sud *et al*. concluding that an extended hospital LOS was associated with increased rates of all types of readmission, except for hospitalisation after heart failure, where a short LOS was associated with increased rates of readmission for cardiovascular disease and heart failure (65).

For peer review only - http://bmjopen.bmj.com/site/about/guidelines.xhtml

Our results indicated a significant positive association between the number of a patient's medical conditions and the 30-day hospital readmission rate, confirming other recent retrospective hospital register studies (66, 67). More specifically, older patients with impaired mobility showed an increased risk of hospital readmission. This result was not surprising, bearing in mind that although these older patients were discharged home—and not to a nursing home—after their hospital stay, their health status might nevertheless require future readmission. Indeed, this corroborated publications about older patients discharged after orthopaedic treatment or who had been initially admitted for heart failure, myocardial infarction or pneumonia, but also presented with impaired mobility (68, 69).

Cognitive impairment was not associated with increased 30-day hospital readmission rates, in line with findings from the systematic review by Pickens *et al*., which pointed out that dementia had a modest impact on readmission rates (70). It was no surprise that inpatients hospitalised for cancer faced a high risk of readmission, corroborating prior studies by Buhenn *et al*., Chang *et al*. and Butcher (71-73).

PP significantly increased the 30-day hospital readmission rate, but this result was based on the average number of drugs prescribed to the sample of readmitted patients versus those not readmitted. Although PP was confirmed as a strong determinant of 30-day hospital readmission in publications by Leendertse *et al*. (74, 75), our results showed a progressive linear relationship between PP and readmission rate, and this should be interpreted with caution. Despite our results and other publications and research underlining the challenge of PP among multimorbid older patients, there is no overall consensus about the best way to deal with the broad general relationship between PP and hospital readmission (76).

Our advanced statistical analysis demonstrated that some specific drugs and the concomitant use of specific drug combinations were significantly associated with 30-day readmission risk, although this was not unexpected and has been confirmed in previous publications (37, 77). Mostly in line with the research findings of Zhang *et al*., drugs including hormones, antineoplastics, immunosuppressors, neoplastic antibiotics and bacterial vaccines were substantial risk factors for hospital readmission (7).

In summary, extended hospital LOS, functional impairments, medical conditions and drugs have been demonstrated to be determinants of 30-day hospital readmission, although not all of them have clinically or pharmacologically relevant interpretations or explanations. Further research involving large samples is needed, notably to explore the drug–drug interactions with the highest risk of hospital readmissions. Statistical predictions of potential drug–drug interactions provide important information for modeling drug combinations and identifying pairs of drugs whose combination creates an exaggerated response (9). As the association between the number of drugs and the risk of hospital readmission was linear, more advanced inferential statistics would be needed to clarify a cut-off point for the mean number of drugs that would significantly increase the readmission rate. In addition, problems involving adherence to prescriptions, social support networks, and stronger or weaker primary health-care structures can all influence hospital readmission rates (39). According to some publications, nearly 70% of people aged over 65 make mistakes with their drugs (78, 79). Information about drug adherence, drug underuse and overuse, drug changes and deprescription by family physicians, as well as medication management at home, would contribute to a more comprehensive understanding of disease- and drug-related 30-day hospital readmissions.

For peer review only - http://bmjopen.bmj.com/site/about/guidelines.xhtml

Finally, it would be interesting to explore the risks of readmission according to different hospital wards. As psychiatric conditions are a frequent cause of rehospitalisation (80), it would be relevant for future research to explore registries from adult psychiatry departments and investigate the hospital readmission risks faced by their inpatients.

Strengths and Limitations

This study's main strength was its use of data recorded in a comprehensive register. We consider this retrospective study useful for clinical practice and future research because a whole series of sociodemographic and clinical parameters, medical conditions and prescribed drugs were used to predict the probability of hospital readmission. Using both bivariate and multivariate analyses enabled an evaluation of the data's longitudinal nature.

Our study had several limitations, nevertheless. The design did not allow us to identify hospitalisations and readmissions lost-to-follow-up and to adjust our data for death outside the hospital. We were also unable to identify unnecessary hospitalisations or any bias towards hospitalisation rather than another health-care solution for older inpatients. Our dataset could not inform us about whether older inpatients had been first admitted to another hospital or were subsequently readmitted elsewhere during the study period. Because the reasons for hospital admission are not chosen from a list but are entered into the register as free descriptive text, these factors were not part of our dataset, and the study was unable to explore the reasons for an admission's impact on 30-day rehospitalisation. Another limitation was the study's lack of formal screening methods to explain ADEs in detail, and it was impossible to distinguish between elective and urgent hospitalisations. Although the study considered statistical associations between drugs and rehospitalisations, it did not use clinically diagnosed drug–drug interactions. Finally, we were unable to consider any potential causality between PP and hospital readmission.

Conclusions

Hospital length of stay, medical conditions, functional impairments and prescribed drugs were all critical factors in predicting hospital readmissions, thus affirming our hypotheses. Readmission patterns are complex and poorly understood because older patients often present with multiple chronic conditions, functional impairments and complex drug prescriptions. Hospital readmission is an under-investigated topic deserving of additional, well-conducted, predictive research exploiting accurate longitudinal data from large samples.

Acknowledgments

The authors thank the partner hospital, including the hospital's data warehouse, for its valuable contributions. This research was developed, in part, using grants from the Swiss National Science Foundation and the School of Health Sciences of the University of Applied Sciences and Arts Western Switzerland (HES-SO) Valais/Wallis. The funders had no role in the design and conduct of the study, the collection, management, analysis and interpretation of the data, the preparation, review or approval of the manuscript, or the decision to submit the manuscript for publication.

Authors Contributions

For peer review only

BW, FP, and HV had the original idea. BW, ZT, SdG, MMM and HV provided conceptual and methodological expertise to the study design and BW, FP, ZT, SdG, CMM, AvG and HV to data analysis and interpretation. BW, FP, and HV were major contributors to writing the manuscript. All authors read, edited, and approved the final manuscript.

Funding

This study was supported by the Swiss National Science Foundation via grant number 407440_183434/1.

Competing interest

The authors report no conflicts of interest surrounding this work.

Ethics approval and patient consent

Ethical approval was obtained from the Human Research Ethics Committee of the Canton of Vaud (CER-VD, 2018-02196), thus permitting our partner hospital's data warehouse to provide the appropriate dataset. Given the retrospective data source, obtaining consent from the patients concerned was impossible or posed disproportionate difficulties. The present study respects the legal requirements for research projects involving data re-use without consent, as set out in Art. 34 from the Swiss Human Research Act (HTA).

Data sharing statement

As part of the Data Use Agreement, authors are not allowed to provide raw data. Upon a reasonable request, the corresponding author will provide statistical programming code used to generate results.

Word Count: 4,158

Figure 1. Relationship between 30-day readmission rate and the number of prescribed drugs at discharge.

References

1. Rayan-Gharra N, Rn ES, Tadmor B, Flaks-Manov N, Balicer RD. Patients' ratings of the in-hospital discharge briefing and post-discharge primary care follow-up: the association with 30-day readmissions. Patient Education and Counseling. 2019.
2. Kabue S, Greene J, Kipnis P, Lawson B, Rinetti-Vargas G, Liu V, et al. The Impact of Pharmacy-specific Predictors on the Performance of 30-Day Readmission Risk Prediction Models. Med Care. 2019;57(4):295-9.
3. Jencks SF, Williams MV, Coleman EA. Rehospitalizations among Patients in the Medicare Fee-for-Service Program. New England Journal of Medicine. 2009;360(14):1418-28.
4. Covinsky KE, Palmer RM, Fortinsky RH, Counsell SR, Stewart AL, Kresevic DM. Loss of independence in activities of daily living in older adults hospitalised with medical illness: increased vulnerability with age. JAGS. 2003;51.
5. Hansen LO, Young RS, Hinami K, Leung A, Williams MV. Interventions to reduce 30-day rehospitalization: a systematic review. Ann Intern Med. 2011;155(8):520-8.
6. Davies EC, Green CF, Mottram DR, Rowe PH, Pirmohamed M. Emergency re-admissions to hospital due to adverse drug reactions within 1 year of the index admission. British Journal of Clinical Pharmacology. 2010;70(5):749-55.

For peer review only

For peer review only - http://bmjopen.bmj.com/site/about/guidelines.xhtml

7. Zhang M, Holman CDAJ, Price SD, Sanfilippo FM, Preen DB, Bulsara MK. Comorbidity and repeat admission to hospital for adverse drug reactions in older adults: retrospective cohort study. BMJ. 2009;338:a2752.
8. Bonnet-Zamponi D, d'Arailh L, Konrat C, Delpierre S, Lieberherr D, Lemaire A, et al. Drug-Related Readmissions to Medical Units of Older Adults Discharged from Acute Geriatric Units: Results of the Optimization of Medication in AGEd Multicenter Randomized Controlled Trial. Journal of the American Geriatrics Society. 2013;61(1):113-21.
9. Blanc A-L, Fumeaux T, Stirnemann J, Dupuis Lozeron E, Ourhamoune A, Desmeules J, et al. Development of a predictive score for potentially avoidable hospital readmissions for general internal medicine patients. PLOS ONE. 2019;14(7):e0219348.
10. Stevenson JM, Davies JG, Martin F, Ali K, Rajkumar C, Schiff R. Is medication related harm as a cause of readmission associated with the indicators of frailty? Age and Ageing. 2018;47:ii19.
11. Brunner-La Rocca H-P, Peden CJ, Soong J, Holman PA, Bogdanovskaya M, Barclay L. Reasons for readmission after hospital discharge in patients with chronic diseases—Information from an international dataset. PloS one. 2020;15(6):e0233457.
12. Dharmarajan K, Hsieh AF, Lin Z, Bueno H, Ross JS, Horwitz LI, et al. Hospital readmission performance and patterns of readmission: retrospective cohort study of Medicare admissions. Bmj. 2013;347.
13. Arora S, Patel P, Lahewala S, Patel N, Patel NJ, Thakore K, et al. Etiologies, trends, and predictors of 30-day readmission in patients with heart failure. The American journal of cardiology. 2017;119(5):760-9.
14. Shams I, Ajorlou S, Yang K. A predictive analytics approach to reducing 30-day avoidable readmissions among patients with heart failure, acute myocardial infarction, pneumonia, or COPD. Health care management science. 2015;18(1):19-34.
15. Krumholz HM, Wang K, Lin Z, Dharmarajan K, Horwitz LI, Ross JS, et al. Hospital-readmission risk—isolating hospital effects from patient effects. New England Journal of Medicine. 2017;377(11):1055-64.
16. Gruneir A, Fung K, Fischer HD, Bronskill SE, Panjwani D, Bell CM, et al. Care setting and 30-day hospital readmissions among older adults: a population-based cohort study. CMAJ. 2018;190(38):E1124-E33.
17. Marengoni A, Angleman S, Melis R, Mangialasche F, Karp A, Garmen A, et al. Aging with multimorbidity: A systematic review of the literature. Ageing Research Reviews. 2011;10:430-9.
18. Valderas JM, Starfield B, Sibbald B, Salisbury C, Rloand M. Defining comorbidity: implications for understanding health and health services. Annals Of Family Medicine. 2009;7:357-63.
19. Maher RL, Hanlon J, Hajjar ER. Clinical consequences of polypharmacy in elderly. Expert Opinion on Drug Safety. 2014;13:57-65.
20. Masnoon N, Shakib S, Kalisch-Ellett L, Caughey GE. What is polypharmacy? A systematic review of definitions. BMC Geriatrics. 2017;17:1-10.
21. Wastesson JW, Morin L, Tan ECK, Johnell K. An update on the clinical consequences of polypharmacy in older adults: a narrative review. Expert Opin Drug Saf. 2018;17(12):1185-96.
22. Rieckert A, Trampisch US, Klaaßen-Mielke R, Drewelow E, Esmail A, Johansson T, et al. Polypharmacy in older patients with chronic diseases: a cross-sectional analysis of factors associated with excessive polypharmacy. BMC Family Practice. 2018;19(1):113.
23. Magill SS, Edwards JR, Bamberg W, Beldavs ZG, Dumyati G, Kainer MA, et al. Multistate point-prevalence survey of health care-associated infections. N Engl J Med. 2014;370(13):1198-208.

For peer review only - http://bmjopen.bmj.com/site/about/guidelines.xhtml

24. Coleman EA, Smith JD, Raha D, Min SJ. Posthospital medication discrepancies: prevalence and contributing factors. Arch Intern Med. 2005;165(16):1842-7.
25. Ferreri SP, Hughes TD, Snyder ME. Medication Therapy Management: Current Challenges. Integr Pharm Res Pract. 2020;9:71-81.
26. Roux P, Verloo H, Santiago-Delefosse M, Pereira F. The spatial dimensions of medication management by home-dwelling older adults after hospital discharge. Health & Place. 2019;60:102230.
27. El Morabet N, Uitvlugt EB, van den Bemt BJF, van den Bemt P, Janssen MJA, Karapinar-Çarkit F. Prevalence and Preventability of Drug-Related Hospital Readmissions: A Systematic Review. J Am Geriatr Soc. 2018;66(3):602-8.
28. Forster AJ, Murff HJ, Peterson JF, Gandhi TK, Bates DW. The incidence and severity of adverse events affecting patients after discharge from the hospital. Ann Intern Med. 2003;138(3):161-7.
29. Yeo I, Cheung JW, Feldman DN, Amin N, Chae J, Wong SC, et al. Assessment of Hospital Readmission Rates, Risk Factors, and Causes After Cardiac Arrest: Analysis of the US Nationwide Readmissions Database. JAMA Network Open. 2019;2(9):e1912208-e.
30. Hauviller L, Eyvrard F, Garnault V, Rousseau V, Molinier L, Montastruc JL, et al. Hospital re-admission associated with adverse drug reactions in patients over the age of 65 years. European Journal of Clinical Pharmacology. 2016;72(5):631-9.
31. Davies EC, Green CF, Mottram DR, Rowe PH, M P. Emergency re-admissions to hospital due to adverse drug reactions within 1 year of the index admission. Br J Clin Pharmacol. 2010;70:749–55.
32. Samoy LJ, Zed PJ, Wilbur K, Balen RM, Abu-Laban RB, Roberts M. Drug-related hospitalizations in a tertiary care internal medicine service of a Canadian hospital: a prospective study. Pharmacotherapy. 2006;26(11):1578-86.
33. Teymoorian SS, Dutcher D, Woods M. ASSOCIATION BETWEEN POSTDISCHARGE ADVERSE DRUG REACTIONS AND 30-DAY HOSPITAL READMISSION IN PATIENTS AGED 80 AND OLDER. Journal of the American Geriatrics Society. 2011;59(5):948-9.
34. WHO. ADHERENCE TO LONG-TERM THERAPIES: Evidence for action. Geneva: World Health Organisation; 2003.
35. Brahma DK, Wahlang JB, Marak MD, Ch Sangma M. Adverse drug reactions in the elderly. J Pharmacol Pharmacother. 2013;4(2):91-4.
36. Davies EA, O'Mahony MS. Adverse drug reactions in special populations - the elderly. Br J Clin Pharmacol. 2015;80(4):796-807.
37. Moura CS, Tavares LS, Acurcio Fde A. [Hospital readmissions related to drug interactions: a retrospective study in a hospital setting]. Rev Saude Publica. 2012;46(6):1082-9.
38. Spinks JM, Kalisch Ellett LM, Spurling G, Theodoros T, Williamson D, Wheeler AJ. Adaptation of potentially preventable medication-related hospitalisation indicators for indigenous populations in Australia using a modified Delphi technique. BMJ Open. 2019;9(11):e031369.
39. Rosen OZ, Fridman R, Rosen BT, Shane R, Pevnick JM. Medication adherence as a predictor of 30-day hospital readmissions. Patient Prefer Adherence. 2017;11:801-10.
40. Pellegrin KL, Lee E, Uyeno R, Ayson C, Goo R. Potentially preventable medication-related hospitalizations: A clinical pharmacist approach to assessment, categorization, and quality improvement. Journal of the American Pharmacists Association. 2017;57(6):711-6.
41. Pereira F, Roux P, Santiago-Delefosse M, von Gunten A, Wernli B, Martins MM, et al. Optimising medication management for polymedicated home-dwelling older adults with multiple chronic conditions: a mixed-methods study protocol. BMJ Open. 2019;9(10):e030030.

For peer review only

For peer review only - http://bmjopen.bmj.com/site/about/guidelines.xhtml

42. Benchimol EI, Smeeth L, Guttmann A, Harron K, Moher D, Petersen I, et al. The REporting of studies Conducted using Observational Routinely-collected health Data (RECORD) Statement. PLOS Medicine. 2015;12(10):e1001885.

43. Surgical interventions clasification S. Swiss classification of surgical interventions (CHOP). 2016.

44. Parker S, Prince A, Thomas L, Song H, Milosevic D, Harris MF. Electronic, mobile and telehealth tools for vulnerable patients with chronic disease: a systematic review and realist synthesis. BMJ Open. 2018;8(8).

45. Olsen J. Register-based research: some methodological considerations. Scandinavian journal of public health. 2011;39(3):225-9.

46. Taushanov Z, Verloo H, Wernli B, Di Giovanni S, von Gunten A, F P. Transforming a patient registry into a customised dataset for the advanced statistical analysis of health risk factors and for medication-related hospitalisation research: a retrospective hospital patient registry study. JMIR Medical Informatics. 2021.

47. Andrade C. Age as a variable: Continuous or categorical? Indian J Psychiatry. 2017;59(4):524-5.

48. Gerka A LC, Pfingsthorn M, Eichelberg M, Muller S, Stolle C, Hein A, editor A Clustering-based Approach to Determine a Standardized Statistic for Daily Activities of Elderly Living Alone. . Proceedings of the 12th International Joint Conference on Biomedical Engineering Systems and Technologies; 2019: SCITEPRESS – Science and Technology Publications, Lda.

49. Trevithick L, Painter J, Keown P. Mental health clustering and diagnosis in psychiatric in-patients. BJPsych bulletin. 2015;39(3):119-23.

50. Delil S, Çelik RN, San S, Dundar M. Clustering patient mobility patterns to assess effectiveness of health-service delivery. BMC health services research. 2017;17(1):1-14.

51. WHO. The Anatomical Therapeutic Chemical Classification System with Defined Daily Doses (ATC/DDD) Geneva: World Health Organization; 2014 [24 March 2020]. Available from: http://www.who.int/classifications/atcddd/en/.

52. Haider SI, Johnell K, Thorslund M, Fastbom J. Trends in polypharmacy and potential drug-drug interactions across educational groups in elderly patients in Sweden for the period 1992 - 2002. Int J Clin Pharmacol Ther. 2007;45(12):643-53.

53. Goldstein H. Nonlinear Multilevel Models, with an Application to Discrete Response Data. Biometrika. 1991;78(1):45-51.

54. Linkens AEMJH, Milosevic V, van der Kuy PHM, Damen-Hendriks VH, Mestres Gonzalvo C, Hurkens KPGM. Medication-related hospital admissions and readmissions in older patients: an overview of literature. International Journal of Clinical Pharmacy. 2020.

55. Berry JG, Gay JC, Joynt Maddox K, Coleman EA, Bucholz EM, O'Neill MR, et al. Age trends in 30 day hospital readmissions: US national retrospective analysis. BMJ. 2018;360:k497.

56. Kongkaew C, Hann M, Mandal J, Williams SD, Metcalfe D, Noyce PR, et al. Risk factors for hospital admissions associated with adverse drug events. Pharmacotherapy. 2013;33(8):827-37.

57. Thunander Sundbom L, Bingefors K. Women and men report different behaviours in, and reasons for medication non-adherence: a nationwide Swedish survey. Pharmacy practice. 2012;10(4):207-21.

58. Manteuffel M, Williams S, Chen W, Verbrugge RR, Pittman DG, Steinkellner A. Influence of patient sex and gender on medication use, adherence, and prescribing alignment with guidelines. J Womens Health (Larchmt). 2014;23(2):112-9.

59. Thunander Sundbom L, Hedborg K. Association between prescribed antidepressants and other prescribed drugs differ by gender: a nationwide register-based study in Sweden. Nord J Psychiatry. 2019;73(1):73-9.

For peer review only

For peer review only - http://bmjopen.bmj.com/site/about/guidelines.xhtml

60. Silverstein MD, Qin H, Mercer SQ, Fong J, Haydar Z. Risk Factors for 30-Day Hospital Readmission in Patients ≥65 Years of Age. Baylor University Medical Center Proceedings. 2008;21(4):363-72.
61. Schwab C, Hindlet P, Sabatier B, Fernandez C, Korb-Savoldelli V. Risk scores identifying elderly inpatients at risk of 30-day unplanned readmission and accident and emergency department visit: a systematic review. BMJ Open. 2019;9(7):e028302.
62. Jain S, Khera R, Mortensen EM, Weissler JC. Readmissions of adults within three age groups following hospitalization for pneumonia: Analysis from the Nationwide Readmissions Database. PLOS ONE. 2018;13(9):e0203375.
63. Hasegawa K, Gibo K, Tsugawa Y, Shimada YJ, Camargo CA, Jr. Age-Related Differences in the Rate, Timing, and Diagnosis of 30-Day Readmissions in Hospitalized Adults With Asthma Exacerbation. Chest. 2016;149(4):1021-9.
64. Shebeshi DS, Dolja-Gore X, Byles J. Unplanned Readmission within 28 Days of Hospital Discharge in a Longitudinal Population-Based Cohort of Older Australian Women. International Journal of Environmental Research and Public Health. 2020;17(9):3136.
65. Sud M, Yu B, Wijeysundera HC, Austin PC, Ko DT, Braga J, et al. Associations Between Short or Long Length of Stay and 30-Day Readmission and Mortality in Hospitalized Patients With Heart Failure. JACC: Heart Failure. 2017;5(8):578-88.
66. Donzé J, Lipsitz S, Bates DW, Schnipper JL. Causes and patterns of readmissions in patients with common comorbidities: retrospective cohort study. Bmj. 2013;347:f7171.
67. Hijazi HH, Alyahya MS, Hammouri HM, Alshraideh HA. Risk assessment of comorbidities on 30-day avoidable hospital readmissions among internal medicine patients. J Eval Clin Pract. 2017;23(2):391-401.
68. Falvey JR, Bade MJ, Hogan C, Forster JE, Stevens-Lapsley JE. Preoperative Activities of Daily Living Dependency is Associated With Higher 30-Day Readmission Risk for Older Adults After Total Joint Arthroplasty. Clin Orthop Relat Res. 2020;478(2):231-7.
69. Greysen SR, Stijacic Cenzer I, Auerbach AD, Covinsky KE. Functional impairment and hospital readmission in Medicare seniors. JAMA Intern Med. 2015;175(4):559-65.
70. Pickens S, Naik AD, Catic A, Kunik ME. Dementia and Hospital Readmission Rates: A Systematic Review. Dementia and Geriatric Cognitive Disorders Extra. 2017;7(3):346-53.
71. Chiang LY, Liu J, Flood KL, Carroll MB, Piccirillo JF, Stark S, et al. Geriatric assessment as predictors of hospital readmission in older adults with cancer. J Geriatr Oncol. 2015;6(4):254-61.
72. Butcher L. Oncologists Seek to Understand, Address Hospital Readmissions. Oncology Times. 2016;38(6):1,9-10.
73. Burhenn P, Sun C-L, Scher KS, Hsu J, Pandya P, Chui C-Y, et al. Predictors of hospital readmission among older adults with cancer. J Geriatr Oncol. 2020;11(7):1108-14.
74. Leendertse AJ, Egberts ACG, Stoker LJ, van den Bemt PMLA, Group HS. Frequency of and Risk Factors for Preventable Medication-Related Hospital Admissions in the Netherlands. Archives of Internal Medicine. 2008;168(17):1890-6.
75. Leendertse AJ, Van Den Bemt PM, Poolman JB, Stoker LJ, Egberts AC, Postma MJ. Preventable hospital admissions related to medication (HARM): cost analysis of the HARM study. Value Health. 2011;14(1):34-40.
76. Garfinkel D, Bilek A. Inappropriate medication use and polypharmacy in older people. BMJ. 2020;369:m2023.
77. Tesfaye WH, Peterson GM, Castelino RL, McKercher C, Jose MD, Wimmer BC, et al. Medication Regimen Complexity and Hospital Readmission in Older Adults With Chronic Kidney Disease. Annals of Pharmacotherapy. 2019;53(1):28-34.
78. Fialová D, Onder G. Medication errors in elderly people: contributing factors and future perspectives. British journal of clinical pharmacology. 2009;67(6):641-5.

For peer review only - http://bmjopen.bmj.com/site/about/guidelines.xhtml

79. Lavan AH, Gallagher PF, O'Mahony D. Methods to reduce prescribing errors in elderly patients with multimorbidity. Clinical interventions in aging. 2016;11:857-66.
80. Cook JA, Burke-Miller JK, Jonikas JA, Aranda F, Santos A. Factors associated with 30-day readmissions following medical hospitalizations among Medicaid beneficiaries with schizophrenia, bipolar disorder, and major depressive disorder. Psychiatry Research. 2020;291:113168.

Table 1. Sociodemographic and hospitalisation data for inpatient stays by polymedicated, home-dwelling adults aged 65 or more (n = 13,802)

**Variables**	**Inpatient stays by polymedicated, home-dwelling adults aged 65 or more (n = 13,802)**
Sex	
Stays by men (%)	7,834 (56.8)
Stays by women (%)	5,968 (43.2)
Age at discharge (years)	
Mean inpatient age at discharge (SD)	77.77 (7.48)
Min–Max	65–106
Med [IQR 25–75]	77.00 [68.00–80.00]
65–69 (%)	2,226 (16.1)
70–79 (%)	5,811 (42.1)
80–89 (%)	4,845 (35.1)
90 and more (%)	920 (6.7)
Year of discharge	
2015 (%)	3,501 (25.4)
2016 (%)	3,318 (24.0)
2017 (%)	3,530 (25.6)
2018 (%)	3,453 (25.0)
Length of stay (days)	
Mean (SD)	8.44 (7.58)
Min–Max	1–149
Med [IQR 25-75]	7 [4–11]
Number of ICDs-10	
Mean (SD)	4.58 (0.92)
Min–Max	1–5
Med [IQR 25–75]	5 [5–5]
Principal ICD-10 diagnosis	
Circulatory (%)	3,336 (24.2)
Infectious (%)	404 (2.9)
Respiratory (%)	1,444 (10.5)
Trauma (%)	1,043 (7.6)
Tumours (%)	1,505 (10.9)
Number of CHOP surgical procedures	
Mean (SD)	1.83 (1.76)
Min–Max	0– 5
Med [IQR 25–75]	1 [0–3]
Number of medicines prescribed at hospital discharge	
Mean (SD)	8.95 (3.24)
Min–Max	5-30
Med [IQR 25–75]	8 [7.50–16.00]

For peer review only - http://bmjopen.bmj.com/site/about/guidelines.xhtml

Table 2. 30-day hospital readmission risks at different periods for different age groups (N = 13,802 readmissions).

Variables	30-day hospital readmission	*p*-value
Complete sample	7.8%	
Sex		*
Women vs men	7.3% vs 8.2%	
Year-end age, in years		NS
65–69	7.5%	
70–79	7.6%	
80–89	8.4%	
≥ 90	6.4%	
Mobility cluster:		NS
Preserved mobility vs impaired mobility	7.6% vs 8.5%	
Activities in daily living (ADL):		NS
Full ADL ability vs impaired ADL	7.8% vs 7.2%	
Cognitive status:		NS
Preserved cognitive status vs cognitive impairment	7.8% vs 7.9%	
ICD-10 diagnosis: circulatory problems		**
No vs Yes	8.2% vs 6.5%	
ICD-10 diagnosis: infection		NS
No vs Yes	7.7% vs 9.9%	
ICD-10 diagnosis: respiratory problems		NS
No vs Yes	7.8% vs 8.0%	
ICD-10 diagnosis: trauma		**
No vs Yes	8.0% vs 5.8%	
ICD-10 diagnosis: tumour		***
No vs Yes	6.9% vs 15.1%	
Number of ICD-10 conditions		***
1	1.5%	
2	4.9%	
3	3.6%	
4	4.8%	
5	8.8%	
Number of surgical procedures (CHOP)		*
0	7.7%	
1	7.8%	
2	7.0%	
3	7.3%	
4	7.1%	
5	9.7%	
Year of discharge: 2015–2018		NS
2015	8.3%	
2016	8.0%	
2017	8.0%	
2018	6.8%	

Note. * = $p < 0.05$; ** = $p < 0.01$; *** = $p < 0.001$; NS = non-significant

For peer review only

For peer review only - http://bmjopen.bmj.com/site/about/guidelines.xhtml

678 Table 3. 30-day hospital readmissions for different classes of drugs based on the ATC (N = 13,802).

Drug class	30-day readmission with NO drugs in this class	30-day readmission with drugs in this class	*p*-value
First level, anatomical main group			
Blood and blood-forming organ drugs (B)	7.1%	8.0%	NS
Dermatologicals (D)	7.7%	9.4%	NS
Genitourinary system and sex hormones (G)	7.7%	8.3%	NS
Systemic hormonal preparations, excluding sex hormones and insulins (H)	7.4%	9.5%	***
Anti-infectives for systemic use (J)	8.0%	7.2%	NS
Antineoplastic and immunomodulating agents (L)	7.6%	12.6%	***
Drugs for the musculo-skeletal system (M)	8.0%	6.5%	*
Antiparasitic products, insecticides, and repellents (P)	7.8%	6.6%	***
Respiratory system drugs (R)	7.4%	9.9%	***
Sensory organ drugs (S)	7.8%	8.4%	NS
Second level, therapeutic subgroup			
Stomatological preparations (A01)	7.8%	12.2%	NS
Drugs for acid-related disorders (A02)	7.0%	8.5%	***
Drugs for functional gastrointestinal disorders (A03)	7.4%	13.4%	***
Antiemetics and antinauseants (A04)	7.7%	27.7%	***
Bile and liver therapy drugs (A05)	7.8%	14.3%	NS
Drugs for constipation (A06)	7.3%	10.8%	***
Antidiarrhoeals, intestinal anti-inflammatory/anti-infective agents (A07)	7.7%	12.9%	***
Digestives, including enzymes (A09)	7.8%	10.0%	NS
Drugs used in diabetes (A10)	7.4%	9.5%	***
Vitamins (A11)	7.5%	9.9%	***
Mineral supplements (A12)	7.4%	8.8%	**
Other alimentary tract and metabolism products (A16)	7.8%	6.3%	NS
Cardiac therapy (C01)	7.6%	8.9%	NS
Antihypertensives (C02)	7.7%	14.1%	***
Diuretics (C03)	7.2%	9.8%	***
Peripheral vasodilators (C04)	7.8%	15.2%	NS
Vasoprotective drugs (C05)	7.8%	9.8%	NS
Beta-blocking agents (C07)	7.1%	8.6%	***
Calcium channel blockers (C08)	7.7%	8.6%	NS
Agents acting on the renin-angiotensin system (C09)	8.7%	7.1%	***
Lipid-modifying agents (C10)	8.3%	7.1%	**
Anaesthetics (N01)	7.8%	18.8%	*
Analgaesics (N02)	7.8%	7.8%	NS
Antiepileptics (N03)	7.7%	9.0%	NS
Drugs for Parkinson's disease (N04)	7.8%	6.9%	NS
Psycholeptics (N05)	6.8%	9.3%	***
Psychoanaleptics (N06)	7.8%	7.7%	NS
Other nervous system drugs (N07)	7.9%	5.1%	NS

679 Note. * = $p < 0.05$; ** = $p < 0.01$; *** = $p < 0.001$; NS = non-significant

680

For peer review only - http://bmjopen.bmj.com/site/about/guidelines.xhtml

Table 4. Baseline, multilevel, logistic regression model using 30-day readmission (0 = no; 1 = yes) as the dependent variable associated with independent sociodemographic, LOS, and clinical variables (N = 13,802 observations for 8,878 different inpatients readmitted to hospital).

**Variables**	**Odds Ratio**[3]	***P* > z**	**95% CI** [4]
Sex	1.079	0.285	0.938–1.242
Year-end age, in years	0.999	0.878	0.990–1.009
Hospital length of stay (LOS), in days	1.014	0.000	1.006–1.021
Mobility cluster [1]	1.218	0.015	1.039–1.427
Dependency in the activities of daily living[1]	0.794	0.248	0.537–1.174
Mental health status[1]	0.992	0.966	0.687–1.433
CIM 1 diagnosis: circulatory problems [2]	0.938	0.491	0.783–1.124
CIM 1 diagnosis: infection [2]	1.381	0.078	0.964–1.977
CIM 1 diagnosis: respiratory problems [2]	1.100	0.414	0.875–1.382
CIM 1 diagnosis: trauma [2]	0.847	0.265	0.633–1.134
CIM 1 diagnosis: tumour [2]	2.538	0.000	2.089–3.082
Number of CIM	1.419	0.000	1.282–1.572
Number of CHOP	0.978	0.304	0.938–1.020
Number of drugs	1.043	0.000	1.028–1.058
Year: 2015 to 2018	0.933	0.022	0.880–0.990
Intercept	.	0.027	.

Note. 1: 0 = good state, 1 = impairment; 2: 0 = no, 1 = yes; 3: adjusted Odds ratio; 4:

Table 5. Multilevel logistic regression model results for the drugs prescribed to older patients at discharge home that had significant predictive values (odds ratios) for 30-day hospital readmission (controlled for variables in the baseline model: Table 4) (N = 13,802 observations for 8,878 different inpatients readmitted to hospital).

**Variables**	**Odds ratio**[1]	***p* > z**	**95% CI** [2]
First level, anatomical main group			
Blood and blood-forming organs drugs (B)	1.089	0.041	1.003–1.181
Systemic hormonal preparations, excluding sex hormones and insulins (H)	1.207	0.007	1.052–1.385
Respiratory system drugs (R)	1.146	0.003	1.046–1.254
Second level, therapeutic subgroup			
Drugs for functional gastrointestinal disorders (A03)	1.424	0.001	1.166–1.739
Antiemetics and antinauseants (A04)	3.216	0.000	1.842–5.617
Drugs for constipation (A06)	1.195	0.018	1.031–1.386
Drugs used in diabetes (A10)	1.125	0.021	1.018–1.243
Vitamins (A11)	1.201	0.008	1.049–1.374
Antihypertensives (C02)	1.771	0.000	1.287–2.438

For peer review only - http://bmjopen.bmj.com/site/about/guidelines.xhtml

Diuretics (C03)	1.149	0.024	1.018–1.296
Beta-blocking agents (C07)	1.156	0.040	1.007–1.327
Lipid-modifying agents (C10)	0.841	0.015	0.732–0.967
Psycholeptics (N05)	1.130	0.009	1.031–1.238

Note. 1 = adjusted odds ratio; 2 = CI or Confidence Interval

Table 6. Drugs and drugs interactions from ATC classes A and B with a significant risk of 30-day hospital readmission (controlled for variables in the baseline model: Table 4) (N = 13,802 observations for 8,878 different inpatients readmitted to hospital).

**Variables**	**Odds ratio[1]**	***p* > z**	**95% CI [2]**
First level, anatomical main group			
Blood and blood-forming organ drugs (B)	1.089	0.040	1.004–1.182
Systemic hormonal preparations, excluding sex hormones and insulins (H)	1.210	0.007	1.054–1.390
Respiratory system drugs (R)	1.149	0.003	1.049–1.258
Second level, therapeutic subgroup			
Antiemetics and antinauseants (A04)	3.222	0.000	1.844–5.630
Drugs for functional gastrointestinal disorders (A03)	1.428	0.000	1.169–1744
Beta-blocking agents (C07) and drugs for acid-related disorders (A02)	1.367	0.022	1.046–1.788
Drugs for constipation (A06)	1.199	0.017	1.033–1.392
Agents acting on the renin-angiotensin system (C09)	0.892	0.049	0.796–0.999
Lipid-modifying agents (C10)	0.838	0.013	0.729–0.964

Note. 1: adjusted odds ratio; 2: CI = Confidence Interval

For peer review only

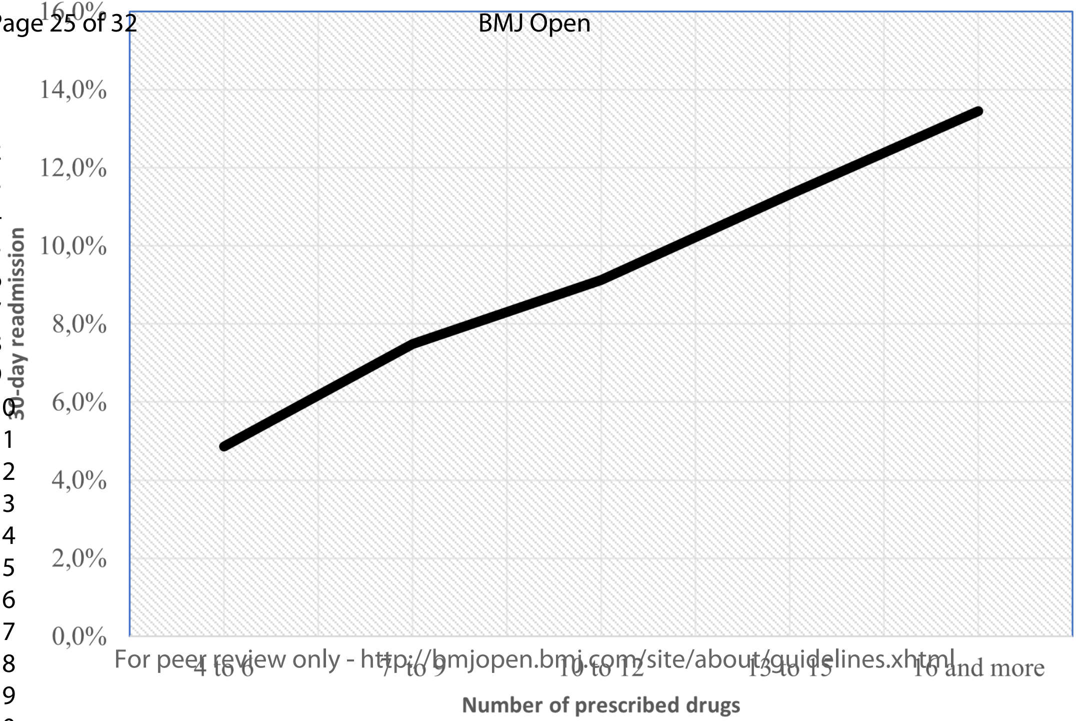
16,0%
14,0%
12,0%
10,0%
8,0%
6,0%
4,0%
2,0%
0,0%
30-day readmission
4 to 6
7 to 9
10 to 12
13 to 15
16 and more
Number of prescribed drugs

Supplementary table. Descriptive statistics of drugs prescribed per hospital stays (N = 13,802 readmissions) at discharge based on the ATC Classification System.

**Drug classes based on the ATC Classification System**	**Min-Max**	**Mean (SD)**
First level, anatomical main group		
Blood and blood forming organs (B)	0–5	1.15 (0.86)
Dermatologicals (D)	0–3	0.04 (0.21)
Genitourinary system and sex hormones (G)	0–4	0.21 (0.47)
Systemic hormonal preparations, excl. sex hormones and insulins (H)	0–4	0.20 (0.46)
Anti-infectives for systemic use (J)	0–4	0.24 (0.47)
Antineoplastic and immunomodulating agents (L)	0–5	0.05 (0.23)
Musculo-skeletal system (M)	0–3	0.15 (0.39)
Antiparasitic products, insecticides and repellents (P)	0–2	0.02 (0.13)
Respiratory system (R)	0–7	0.28 (0.72)
Sensory organs (S)	0–6	0.10 (0.39)
Second level, therapeutic subgroup		
Stomatological preparations (A01)	0–1	0.00 (0.06)
Drugs for acid-related disorders (A02)	0–3	0.56 (0.52)
Drugs for functional gastrointestinal disorders (A03)	0–3	0.07 (0.28)
Antiemetics and antinauseants (A04)	0–1	0.01 (0.08)
Bile and liver therapy (A05)	0–1	0.00 (0.05)
Drugs for constipation (A06)	0–3	0.15 (0.40)
Antidiarrhoeals, intestinal anti-inflammatory/anti-infective agents (A07)	0–2	0.03 (0.18)
Digestives, incl. Enzymes (A09)	0–2	0.02 (0.13)
Drugs used in diabetes (A10)	0–5	0.26 (0.63)
Vitamins (A11)	0–4	0.15 (0.44)
Mineral supplements (A12)	0–3	0.29 (0.51)
Other alimentary tract and metabolism products (A16)	0–1	0.00 (0.05)
Cardiac therapy drugs (C01)	0–4	0.14 (0.42)
Antihypertensives (C02)	0–2	0.02 (0.17)
Diuretics (C03)	0–3	0.27 (0.53)
Peripheral vasodilators (C04)	0–1	0.00 (0.06)
Vasoprotectives (C05)	0–3	0.02 (0.14)
Beta-blocking agents (C07)	0–2	0.46 (0.51)
Calcium channel blockers (C08)	0–2	0.16 (0.37)
Agents acting on the renin-angiotensin system (C09)	0–3	0.64 (0.62)
Lipid modifying agents (C10)	0–3	0.43 (0.52)
Anaesthetics (N01)	0–1	0.00 (0.05)
Analgaesics (N02)	0–7	1.02 (0.91)
Antiepileptics (N03)	0–5	0.11 (0.35)
Drugs for Parkinson’s disease (N04)	0–5	0.04 (0.24)
Psycholeptics (N05)	0–6	0.53 (0.73)
Psychoanaleptics (N06)	0–3	0.20 (0.44)
Other nervous system drugs(N07)	0–3	0.03 (0.19)
**Total number of drugs**	**5–30**	**8.95 (3.24)**

For peer review only

The RECORD statement – checklist of items, extended from the STROBE statement, that should be reported in observational studies using routinely collected health data.

	Item No.	STROBE items	Location in manuscript where items are reported	RECORD items	Location in manuscript where items are reported
**Title and abstract**					
	1	(a) Indicate the study's design with a commonly used term in the title or the abstract (b) Provide in the abstract an informative and balanced summary of what was done and what was found		RECORD 1.1: The type of data used should be specified in the title or abstract. When possible, the name of the databases used should be included.<br><br>RECORD 1.2: If applicable, the geographic region and timeframe within which the study took place should be reported in the title or abstract.<br><br>RECORD 1.3: If linkage between databases was conducted for the study, this should be clearly stated in the title or abstract.	Title<br>Abstract (lines 2-8)<br><br>Line 6<br><br>Lines 118-121<br><br>Not applicable, only one hospital register
**Introduction**					
Background rationale	2	Explain the scientific background and rationale for the investigation being reported			Lines 39-101
Objectives	3	State specific objectives, including any prespecified hypotheses			Lines 101-107
**Methods**					
Study Design	4	Present key elements of study design early in the paper			Lines 110-114
Setting	5	Describe the setting, locations, and relevant dates, including periods of recruitment, exposure, follow-up, and data collection			Lines 117-133

For peer review only

Participants	6	*(a) Cohort study* - Give the eligibility criteria, and the sources and methods of selection of participants. Describe methods of follow-up<br>*Case-control study* - Give the eligibility criteria, and the sources and methods of case ascertainment and control selection. Give the rationale for the choice of cases and controls<br>*Cross-sectional study* - Give the eligibility criteria, and the sources and methods of selection of participants<br><br>*(b) Cohort study* - For matched studies, give matching criteria and number of exposed and unexposed<br>*Case-control study* - For matched studies, give matching criteria and the number of controls per case		RECORD 6.1: The methods of study population selection (such as codes or algorithms used to identify subjects) should be listed in detail. If this is not possible, an explanation should be provided.<br><br>RECORD 6.2: Any validation studies of the codes or algorithms used to select the population should be referenced. If validation was conducted for this study and not published elsewhere, detailed methods and results should be provided.<br><br>RECORD 6.3: If the study involved linkage of databases, consider use of a flow diagram or other graphical display to demonstrate the data linkage process, including the number of individuals with linked data at each stage.	in press/ forthcoming JMIR article: https://www.ncbi.nlm.nih.gov/pubmed/management/validator/909A44E74F70/citations/?start=0<br><br>Not applicable, only one hospital register
Variables	7	Clearly define all outcomes, exposures, predictors, potential confounders, and effect modifiers. Give diagnostic criteria, if applicable.		RECORD 7.1: A complete list of codes and algorithms used to classify exposures, outcomes, confounders, and effect modifiers should be provided. If these cannot be reported, an explanation should be provided.	Lines 152-177 and in press/ forthcoming JMIR article: https://www.ncbi.nlm.nih.gov/pubmed/management/validator/909A44E74F70/citations/?start=0
Data sources/ measurement	8	For each variable of interest, give sources of data and details			Lines 122-128 and

For peer review only

		of methods of assessment (measurement). Describe comparability of assessment methods if there is more than one group			in press/ forthcoming JMIR article: https://www.ncbi.nlm.nih.gov/pubmed/management/validator/909A44E74F70/citations/?start=0
Bias	9	Describe any efforts to address potential sources of bias			Lines 180-200 and in press/ forthcoming JMIR article: https://www.ncbi.nlm.nih.gov/pubmed/management/validator/909A44E74F70/citations/?start=0
Study size	10	Explain how the study size was arrived at			Lines 141-144
Quantitative variables	11	Explain how quantitative variables were handled in the analyses. If applicable, describe which groupings were chosen, and why			Lines 139-150 and in press/ forthcoming JMIR article: https://www.ncbi.nlm.nih.gov/pubmed/management/validator/909A44E74F70/citations/?start=0

For peer review only

Statistical methods	12	(a) Describe all statistical methods, including those used to control for confounding<br>(b) Describe any methods used to examine subgroups and interactions<br>(c) Explain how missing data were addressed<br>(d) *Cohort study* - If applicable, explain how loss to follow-up was addressed<br>*Case-control study* - If applicable, explain how matching of cases and controls was addressed<br>*Cross-sectional study* - If applicable, describe analytical methods taking account of sampling strategy<br>(e) Describe any sensitivity analyses			Lines 180-200
Data access and cleaning methods		..		RECORD 12.1: Authors should describe the extent to which the investigators had access to the database population used to create the study population.<br><br>RECORD 12.2: Authors should provide information on the data cleaning methods used in the study.	Lines 118-122 and in press/ forthcoming JMIR article: https://www.ncbi.nlm.nih.gov/pubmed/management/validator/909A44E74F70/citations/?start=0
Linkage		..		RECORD 12.3: State whether the study included person-level, institutional-level, or other data linkage across two or more databases. The methods of linkage and methods of	in press/ forthcoming JMIR article: https://www.ncbi.nlm.nih.gov/pubm

For peer review only

				linkage quality evaluation should be provided.	ed/management/validator/909A44E74F70/citations/?start=0
**Results**					
Participants	13	(a) Report the numbers of individuals at each stage of the study (*e.g.*, numbers potentially eligible, examined for eligibility, confirmed eligible, included in the study, completing follow-up, and analysed)<br>(b) Give reasons for non-participation at each stage.<br>(c) Consider use of a flow diagram		RECORD 13.1: Describe in detail the selection of the persons included in the study (*i.e.,* study population selection) including filtering based on data quality, data availability and linkage. The selection of included persons can be described in the text and/or by means of the study flow diagram.	Lines 142-143
Descriptive data	14	(a) Give characteristics of study participants (*e.g.*, demographic, clinical, social) and information on exposures and potential confounders<br>(b) Indicate the number of participants with missing data for each variable of interest<br>(c) *Cohort study* - summarise follow-up time (*e.g.*, average and total amount)			Lines 152-177
Outcome data	15	*Cohort study* - Report numbers of outcome events or summary measures over time<br>*Case-control study* - Report numbers in each exposure category, or summary measures of exposure<br>*Cross-sectional study* - Report numbers of outcome events or summary measures			Lines 142-144

For peer review only

Main results	16	(a) Give unadjusted estimates and, if applicable, confounder-adjusted estimates and their precision (e.g., 95% confidence interval). Make clear which confounders were adjusted for and why they were included<br>(b) Report category boundaries when continuous variables were categorized<br>(c) If relevant, consider translating estimates of relative risk into absolute risk for a meaningful time period			Lines 207-285
Other analyses	17	Report other analyses done—e.g., analyses of subgroups and interactions, and sensitivity analyses			Lines 207-285
**Discussion**					
Key results	18	Summarise key results with reference to study objectives			Lines 289-294
Limitations	19	Discuss limitations of the study, taking into account sources of potential bias or imprecision. Discuss both direction and magnitude of any potential bias		RECORD 19.1: Discuss the implications of using data that were not created or collected to answer the specific research question(s). Include discussion of misclassification bias, unmeasured confounding, missing data, and changing eligibility over time, as they pertain to the study being reported.	Lines 369-378
Interpretation	20	Give a cautious overall interpretation of results considering objectives, limitations, multiplicity of analyses, results from similar studies, and other relevant evidence			Lines 294-361

For peer review only

Generalisability	21	Discuss the generalisability (external validity) of the study results			Lines 349-355
**Other Information**					
Funding	22	Give the source of funding and the role of the funders for the present study and, if applicable, for the original study on which the present article is based			Line 399
Accessibility of protocol, raw data, and programming code		..		RECORD 22.1: Authors should provide information on how to access any supplemental information such as the study protocol, raw data, or programming code.	in press/ forthcoming JMIR article: https://www.ncbi.nlm.nih.gov/pubmed/management/validator/909A44E74F70/citations/?start=0

*Reference: Benchimol EI, Smeeth L, Guttmann A, Harron K, Moher D, Petersen I, Sørensen HT, von Elm E, Langan SM, the RECORD Working Committee. The REporting of studies Conducted using Observational Routinely-collected health Data (RECORD) Statement. *PLoS Medicine* 2015; in press.

*Checklist is protected under Creative Commons Attribution (CC BY) license.

For peer review only
